# Unveiling radiobiological traits and therapeutic responses of BRAF^V600E^-mutant colorectal cancer via patient-derived organoids

**DOI:** 10.1186/s13046-025-03349-z

**Published:** 2025-03-11

**Authors:** Peiyuan Mu, Shaobo Mo, Xingfeng He, Hui Zhang, Tao Lv, Ruone Xu, Luoxi He, Fan Xia, Shujuan Zhou, Yajie Chen, Yaqi Wang, Lijun Shen, Juefeng Wan, Lili Huang, Weiqing Lu, Xinyue Liang, Xiaomeng Li, Ping Lu, Junjie Peng, Guoqiang Hua, Kewen Hu, Zhen Zhang, Yan Wang

**Affiliations:** 1 Department of Radiation Oncology, Fudan University Shanghai Cancer Center, Shanghai, 200032 China; 2 Department of Oncology, Shanghai Medical College, Fudan University, Shanghai, 200032 China; 3 Shanghai Clinical Research Center for Radiation Oncology, Shanghai, 200032 China; 4 Shanghai Key Laboratory of Radiation Oncology, Shanghai, 200032 China; 5 Department of Colorectal Surgery, Fudan University Shanghai Cancer Center, Fudan University, Shanghai, 200032 China; 6 Department of Urology Surgery, Fudan University Shanghai Cancer Center, Fudan University, Shanghai, 200032 China; 7 Cancer institute, Fudan University Shanghai Cancer Center, Shanghai, 200032 China; 8 D1Med Technology (Shanghai) Inc, Shanghai, 201802 China

**Keywords:** BRAF^V600E^-mutant, Radiotherapy, Organoid model, Precision treatment, Colorectal cancer

## Abstract

**Background:**

Radiotherapy (RT) is an essential treatment for colorectal cancer (CRC), yet the factors influencing radiosensitivity remain unclear. In the quest to enhance the therapeutic efficacy in CRC, the interplay between genetic mutations and RT sensitivity has emerged as a pivotal yet enigmatic area.

**Methods:**

We harness the fidelity of patient-derived organoids (PDOs) to dissect the molecular landscape of radiosensitivity, with a particular emphasis on BRAF^V600E^ mutations. To further investigate, a cohort of 9 BRAF^V600E^-mutant and 10 BRAF wild-type PDOs is constructed to systematically assess the radiobiological traits of BRAF^V600E^-mutant CRC, including morphology, cell viability, and DNA damage, while also evaluating their responses to chemotherapy and chemoradiotherapy.

**Results:**

Our systematic investigation unveils a profound correlation between BRAF^V600E^ mutation status and radioresistance, which is validated by clinical treatment responses. Intriguingly, BRAF^V600E^-mutant PDOs exhibit reduced sensitivity to conventional chemotherapy, yet demonstrate an enhanced response to combined chemoradiotherapy, characterized by increased apoptosis. The results are validated through in vivo analyses using patient-derived organoid xenograft mouse models and aligned with patient clinical outcomes.

**Conclusions:**

This study outlines the distinct radiobiological profile of BRAF^V600E^-mutant CRC, underscoring the critical role of radiotherapy in comprehensive treatment strategies. This work not only advances our molecular understanding of CRC but also paves the way for precision medicine, offering valuable insights for therapeutic decision-making in the clinical management of BRAF^V600E^-mutant CRC.

**Supplementary Information:**

The online version contains supplementary material available at 10.1186/s13046-025-03349-z.

## Introduction

Colorectal cancer (CRC) remains a significant global health challenge, ranking as the third most common malignant tumor worldwide [1]. The treatment strategies for colorectal cancer mainly encompassing surgical intervention, chemotherapy, and radiotherapy (RT) [2]. RT is essential in treating CRC, especially locally advanced rectal cancer (LARC), where the standard approach includes neoadjuvant chemoradiotherapy (CRT) followed by radical resection. This approach is favored because it enhances locoregional control, disease-free survival (DFS), and the rates of sphincter preservation [3, 4].

Despite its efficacy, the sensitivity of CRC to RT varies significantly among patients, influenced by a range of factors including genetic mutations [5], molecular characteristics [6–8] and tumor microenvironment [9]. Understanding these factors is pivotal for optimizing RT outcomes. Previous studies have identified various genetic markers, such as KRAS status [10, 11], specific KRAS codon mutations [12], TP53 status [13], BRAF and SMAD4 status [14], which can predict CRT efficacy. However, findings are inconsistent and many have not been validated in in vitro and in vivo studies [5].

BRAF mutation is a relatively rare mutation associated with poor overall prognosis, posing a significant clinical challenge [15, 16]. Previous studies have indicated that patients with this mutation tend to exhibit resistance to conventional chemotherapy [17, 18]; however, research on their radiosensitivity is limited. Dan Jiang et al. reported that patients with LARC harboring BRAF mutations exhibited poor response to neoadjuvant CRT [14], whereas other studies yielded inconsistent results [5, 19]. Consequently, whether these patients can benefit from RT and CRT remains to be further investigated. And there is a notable significance of research regarding the role of RT in treating BRAF-mutant CRC.

Patient-derived organoids (PDOs) is an emerging model for cancer research replicating the 3D structure, genetic, and phenotypic characteristics of original tumors. This model enables precise study of tumor biology, drug responses, and resistance mechanisms [20, 21]. Organoids facilitate personalized medicine by predicting patient-specific treatment outcomes and are crucial in advancing cancer research and therapeutic development [22–24].

Therefore, in this study, we used PDO models to investigate biomarkers influencing radiosensitivity in CRC. Combining organoid radiosensitivity assays with whole-exome sequencing (WES) data, we explored the impact of BRAF mutations on radioresistance. Through systematic in vitro and in vivo experiments, we evaluated the sensitivity of BRAF^V600E^-mutant CRC to RT, chemotherapy, and combined chemoradiotherapy. These findings were validated against clinical treatment responses, providing valuable insights and guidance for RT decisions in the clinical management of these patients.

## Materials and methods

The source of all reagents, chemicals, and biological samples used in this study are listed in the Supplementary Materials Key Resource Table.

### Human specimens

All human specimen collection and experiments were reviewed and approved by the Institutional Review Boards of Fudan University Shanghai Cancer Center (1704171-19-2107 A). CRC tissues were obtained from surgical samples of patients who underwent enterectomy or biopsies of patients who underwent anoscopic biopsy at Fudan University Shanghai Cancer Center and LM tissues were obtained from patients undergoing hepatectomy. Clinical information of CRC patients was obtained through the medical records system. The studies were conducted in accordance with the Declaration of Helsinki and Informed consent was obtained from all participants.

### Organoid culture and biobanking

Human CRC organoids culture medium was used to culture CRC organoids and detailed culture conditions are listed in the Supplementary Materials. For organoids culture, fresh medium was changed every 3 days and photographs of organoids were captured using microscopy (Olympus, iX73). For passage of organoids, the mixture of organoid and Matrigel was collected with organoid anti-adherence solution (D1Med, D23025-0050), pipetted 50–100 times and centrifuged at 500 g for 5 min. The organoid was collected and passaged at a 1:3 ratio.

Organoids were biobanked at various passages and for each sample at least 5 vials were frozen for biobanking. Cryopreservative medium (serum free) (CELLBANKERTM 2, ZENOAQ, 170905) was used to freeze organoids. All organoids used in this study were passaged fewer than 10 times.

### Whole-exome sequencing

Organoids in good condition were harvested and frozen, with their DNA extracted using the SDS method and germline DNA was extracted from frozen peripheral blood mononuclear cells. Details regarding whole exome sequencing and analysis are provided in Supporting Information.

### Organoid irradiation

Organoids were seeded in 96-well plates, exposed to X-ray (Raycision, Sharp 100) and photographed every 6 days after radiation (Olympus, iX73). For dose response assays, organoids were subjected to varying radiation dose (0, 4, 8, 12, and 16 Gy), and number of surviving organoids was manually counted. Survival fraction was calculated as [(number of surviving organoids on day 6)/(number of surviving organoids on day 0)]/[(number of organoids in 0 Gy group on day 6)/(number of organoids in 0 Gy group on day 0)] and radiation dose survival curves were fitted to the SHMT model [25]. D_0_ (the dose required to reduce the fraction of surviving cells to 37% of its previous value) was calculated according to previous study [26].

To assess organoid size changes, the area of surviving organoids was measured using Image-Pro Plus 6.0 (Media Cybernetics, Inc.) at day 0, day 12, and day 24 after treatment with 8 Gy of irradiation. For organoids cell viability assay, CellTiter-Glo® 3D Cell viability assay (Promega, G9683) was performed on day 0 and day 6 after 8 Gy irradiation.

### H&E and immunohistochemistry staining

Organoids and tumor tissues were fixed in 4% paraformaldehyde (organoids at 37 °C for 30 min, tissues at 4 °C for 16–18 h), dehydrated, and embedded in paraffin. Sections (4 μm) were deparaffinized, rehydrated, and stained with H&E or subjected to immunohistochemical staining. For antigen retrieval, sections were heated in citrate antigen retrieval solution (Servicebio, G1202), then treated with hydrogen peroxide, and blocked with donkey serum (Solarbio, SL050). Primary antibodies for β-catenin, CDX2, CK20, Ki67, γH2AX, Bcl-2, Cleaved Caspase-3 (see Supplementary Materials for details) were incubated overnight at 4 °C, followed by secondary antibody incubation (Servicebio, G1215, G1216). Images were acquired by microscope (Olympus, BX43F).

### Sanger sequencing based BRAF genotyping of organoids

Genomic DNA of organoids was extracted using the TIANamp Genomic DNA Kit (TIANGEN, #DP304-02) following the protocol for cultured cells. The following primers were used for genotyping: BRAF-Forward: 5’-TAAAAATAGGTGATTTTGGTCTAGCTGC-3’ BRAF-Reverse: 5’-CCAAAAATTTAATCAGTGGAAAAATA-3’. PCR was carried out in a 25 μl volume containing 12.5 μl of 2×Hieff® PCR Master Mix (Yeasen, 10102), 1 μl of each primer of BRAF-F and BRAF-R and 50 ng of DNA. PCR conditions were as follows: 94 °C for 5 min and 35 cycles of 94 °C for 30 s, 60 °C for 30 s, 72 °C for 30 s, and finally 10 min at 72 °C. PCR products were sequenced by Tsingke company.

### Drug treatment of organoids

The organoids were plated in 96-well plates, and when they were in good growth condition, the organoid culture medium was discarded and replaced with a drug-containing medium with 10 μM 5-FU (Selleck, S1209), 10 μM irinotecan (Selleck, S2217), 10 μM oxaliplatin (Selleck, S1224) or the combination of two or three drugs. In the combination treatment groups of 5FU + OX, 5FU + CPT, and 5FU + OX + CPT, the concentrations of 5FU, OX, and CPT are the same as their respective monotherapies. After 3 days the medium was replaced with fresh drug-containing medium. Total drug treatment duration was 6 days. Organoids were imaged by microscope (Olympus, iX73) every 6 days. And the area of surviving organoids was calculated using Image-Pro Plus 6.0 (Media Cybernetics, Inc.). For chemoradiotherapy sensitivity test, a single irradiation of 8 Gy was administered on day 0 according to our previous studies [27, 28].

### Patient clinical information collection and efficacy assessment

The BRAF gene mutations status of the LARC cohort were detected by pathologists using the AmoyDx Human BRAF Gene V600E Mutation Fluorescent PCR Detection Kit or by next-generation sequencing (NGS). Patients were followed up on an outpatient basis or by telephone to obtain survival information. For patients who underwent surgery, pathological tumor regression grade (pTRG) was evaluated according to the American Joint Committee on Cancer (AJCC) Staging Manual (seventh edition) system [29]. Efficacy of chemoradiotherapy and chemotherapy was evaluated by a professional physician and radiologist according to the RECIST guideline (version 1.1) [30].

### Immunofluorescent staining of organoids

For immunofluorescent staining, organoids were seeded onto 24-well culture plates, each well containing a 12 mm diameter coverslip precoated with 50 μl of 50% Matrigel and incubated at 37 °C. Immunostaining was carried out using a standard protocol as previously reported [26]. Organoids were washed with PBS and fixed with 4% paraformaldehyde for 30 min. After permeabilization with 0.2% Triton X-100/PBS on ice, samples were blocked with 1% BSA/PBS for 2 h and incubated overnight at 4 °C with primary antibody for phospho-H2AX-Ser139, CD44 and Ki67 (see Supplementary Materials for details), followed by Alexa-dye–conjugated secondary antibodies for 2 h. After mounting with ProLong Diamond Antifade Mountant with DAPI (ThermoFisher, P36971), fluorescent images were taken by confocal microscope (Leica, STELLARIS 5). Nine consecutive sections spanning 2 μm were imaged and max-projected using Leica LAS X software as previously reported [31].

### Construction and validation of BRAFV600E overexpressing Caco-2 cell line and BRAF knockdown HT29 cell line

For BRAFV600E overexpressing Caco-2 cell line, cDNAs encoding BRAF^V600E^ were cloned into pLVX-EGFP-IRES-puro (Addgene, #128652). For BRAF knockdown HT29 cell line, shRNAs targeting BRAF gene were designed and cloned into pLKO.1 puro (Addgene, #8453). The shRNA sequences are as follows: BRAF-sh1: GAACATATAGAGGCCCTATTG; BRAF-sh2: GTCATCAGAATGCAAGATAAA.

In brief, virus particles were produced in HEK293T cells using PEI transfection. After filtration with a Millex-HV sterile 0.45 μm filter (Merck Millipore) and titration, viruses were added to cells in presence of polybrene (7.5 μg ml^− 1^) (Yeasen, 40804ES76). Medium was replaced 48 h after infection, followed by selection with puromycin (2 μg ml^− 1^) (Yeasen.54752ES08) for 1–2 weeks. Expression of BRAF^V600E^ and downstream genes were confirmed by Western blot.

### Apoptosis analysis

Organoid apoptosis was measured using a PE Annexin V /propidium iodide apoptosis detection kit (BD Pharmingen, 559763) according to the manufacturer’s instruction. Briefly, the organoids were treated with 10 μM 5-FU or 10 μM 5-FU combined with 8 Gy irradiation for 24 h. After treatment, organoids were digested into single cells using TrypLE™ Express (Gibco, 12605-010). Resuspend cells in 1× Binding Buffer and then add 5 μl of PE Annexin V and 5 μl 7-AAD to each sample and incubate for 15 min. The apoptosis rate was detected by flow cytometry (CytoFLEX, Beckman Coulter, Indianapolis, IN, USA) within 1 h and was analyzed by FlowJo version 10 (https://www.flowjo.com/).

### Cell viability assays and combination index calculation

To determine the IC50 and IC70 values, 3000–4000 cells were plated in 96-well plates and treated with various concentrations of the indicated chemicals and radiation for 72 h. Cell viability was assessed using the CCK-8 assay (GlpBio, GK10001) according to the manufacturer’s instructions. For the combination index (CI) analysis, 5FU and BRAF inhibitors were applied at gradient concentrations. CI calculations were performed using CompuSyn software (version 1.0) with the Chou–Talalay method [32].

### Colony formation assay

300-20000 cells were seeded into 6-well plates and cultured overnight. The next day, the culture medium was replaced with either fresh medium or medium containing 5-FU. The medium was refreshed every 5 days. After 10 to 14 days, colonies became clearly visible. The colonies were then fixed with methanol for 15 min, followed by staining with 0.1% crystal violet for 20 min. Then, the colonies were photographed and counted.

### PDOX mouse model and drug/ radiation treatment

For the CRC PDOX mouse model, two 6-week-old female NSG mice were subcutaneously implanted with BRAF^V600E^1 organoids. When reaching 500 mm³, tumors were dissected and re-transplanted into 6-week-old female BALB/c nude mice. Once the tumors reached 100–150 mm³, mice were randomly divided into four groups: Ctrl (PBS + untreated), RT (PBS + 2 Gy radiation), 5FU (5FU 30 mg/kg + untreated), and 5FU + RT (5FU 30 mg/kg + 2 Gy radiation), with 5 mice in each group. 5FU and PBS were administered via intraperitoneal injection, and radiation therapy was applied using a small animal irradiator (Raycision, Sharp 100), targeting only the tumor area while shielding the rest of the body. Treatments were administered every 3 days. The mice were euthanized at the end of the experiment, tumors and blood samples were collected for further analysis. All animals were purchased from GemPharmatech Co., Ltd (Jiangsu, China), and all procedures were approved by the Ethics Committee of Fudan University Shanghai Cancer Center (FUSCC-IACUC-S2023-0057).

### Statistical analysis

Values represent mean ± SD. Student’s t-test, one-way ANOVA, two-way ANOVA and Kaplan–Meier analysis were performed using the GraphPad Prism 9 software or the R software (version 3.6.1, www.r-project.org). All statistics tested 2-sided, and *p* values < 0.05 was regraded statistically significant. *p < 0.05; **p < 0.01; ***p < 0.001; ****p < 0.0001; ns, not significant. Data was plotted using GraphPad Prism 9 or the R software.

## Results

### BRAF mutations correlate with radiotherapy resistance in CRC

**Fig. 1:**
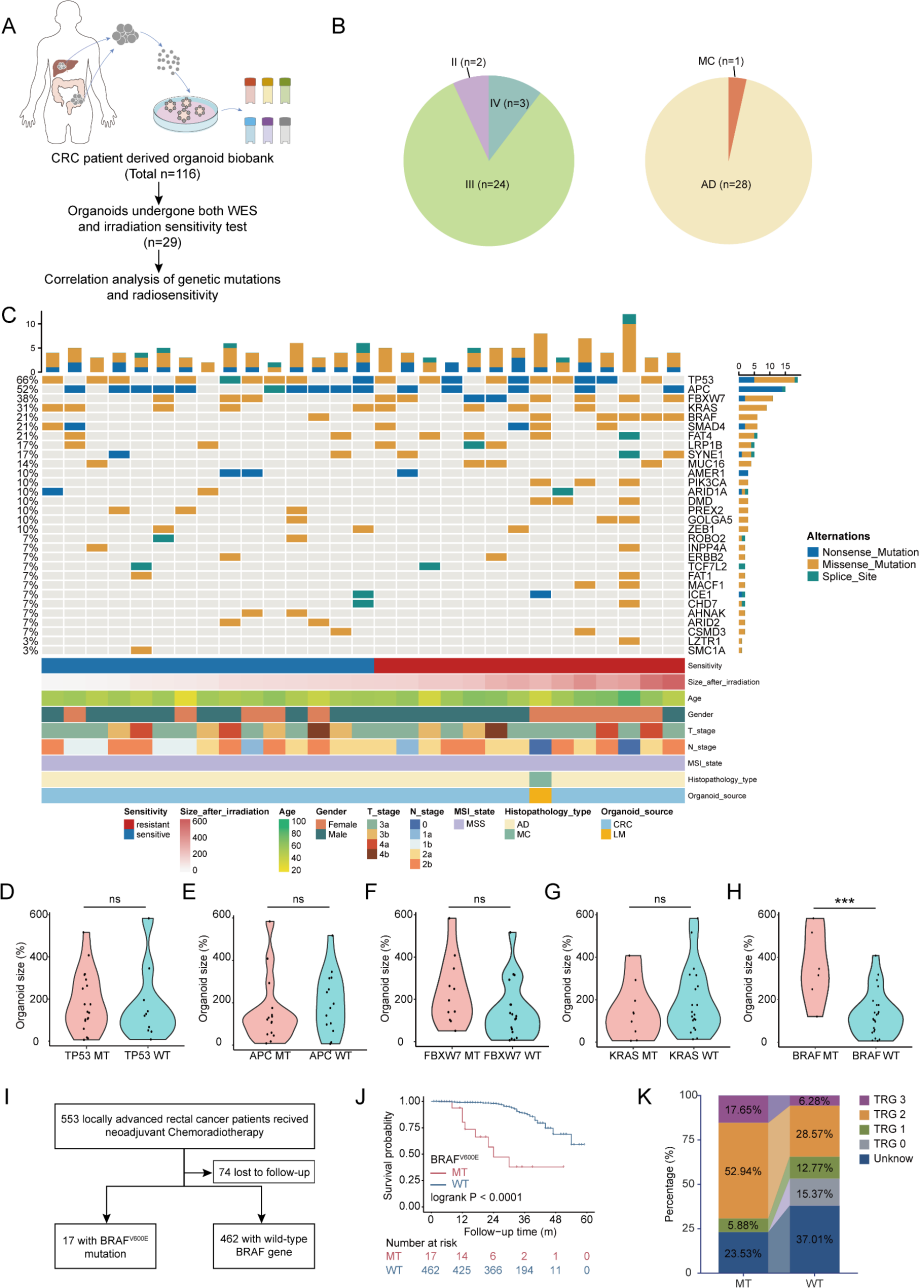
BRAF mutation is associated with radioresistance in colorectal cancer. (**A**) Flowchart for analyzing the relationship between radiation sensitivity and gene mutations based on organoid models. A biobank containing 116 patient-derived organoids was constructed. From these, 29 samples with both radiation therapy sensitivity data and WES data were selected for correlation analysis. (**B**) The tumor stage and histological type distribution of the 29 selected PDOs. (**C**) Overview of somatic mutations in known driver genes found in 29 PDOs. Organoids were grouped based on the size ratio at 24 days after 8 Gy radiation to the area on day 0. Those with a ratio greater than the median (135.03%) were classified as the radiation-resistant group (*n* = 14), while those with a ratio less than or equal to the median were classified as the sensitive group (*n* = 15). **D-H**) The size ratios at 24 days post 8 Gy radiation for organoids with the wild-type (WT) and mutated forms (MT) of the top 5 most frequently mutated genes (TP53, APC, FBXW7, KRAS and BRAF) were analyzed by student’s t-test to compare their radiation sensitivity (BRAF mutated vs. wild-type: 355.3% vs. 132.8%, *p* = 0.0004). **I**) Inclusion flowchart of patients with locally advanced rectal cancer who received neoadjuvant chemoradiotherapy. **J**) Kaplan–Meier analysis of the probability of survival in patients with BRAF^V600E^ mutation and BRAF wild type (Log-rank test, HR: 8.19, 95% CI: 1.24–54.06, *p*<0.0001). **K**) The distribution of TRG after neoadjuvant chemoradiotherapy was compared between patients with BRAF wild-type (WT) and those with BRAF^V600E^ mutation (MT). See also Table S1(Supporting Information)

From 2019 to 2023, a biobank comprising organoids derived from 116 CRC patients was established at Fudan University Shanghai Cancer Center (FUSCC). In order to analyze the relationship between RT sensitivity and genetic mutations, 29 organoids from this biobank, which had both RT sensitivity data and Whole Exome Sequencing (WES) data available, were selected for correlation analysis (WES data and RT sensitivity results for 14 organoids were obtained from our previous study [28]) (Fig. 1A). The 29 selected organoids were all from microsatellite stable (MSS) CRC patients and characterized based on their tumor stage and histological type (Fig. 1B). Among the 29 organoids, stage III tumors constituted the majority with 24 cases (82.76%), followed by 2 cases (6.90%) of stage II tumors and 3 cases (10.34%) of stage IV tumors. Regarding histological classification, 28 cases were adenocarcinomas (AD), representing the vast majority, with 1 case of mucinous adenocarcinoma (MC). This comprehensive characterization provided a detailed context for understanding the genetic landscape and RT sensitivity.

To investigate the potential link between RT sensitivity and genetic mutations, and to identify CRC subtypes resistant to RT, an overview of tumor driven mutations in the 29 selected organoids was conducted. (Fig. 1C). Organoids were categorized into RT-resistant and RT-sensitive groups based on the ratio of organoid size at 24 days post 8 Gy radiation to that at day 0, which had been previously verified to reflect radiosensitivity of CRC organoids and exhibit high consistency with the clinical outcomes of patients [27, 28]. Those with a ratio greater than the median (135.03%) were classified as RT-resistant (*n* = 14), while those with a ratio less than or equal to the median were classified as RT-sensitive (*n* = 15). Detailed clinical information and RT sensitivity results are provided in Table S1. The relationship between the mutation status of the top five frequently mutated genes and RT sensitivity was analyzed using student’s t-tests (Fig. 1D-H). It was observed that only organoids with BRAF mutations exhibited significantly greater RT resistance compared to wild-type organoids (organoid size ratio: 355.3% vs. 132.8%, *p* = 0.0004) (Fig. 1H).

To further validate this finding, a cohort of 553 patients with MSS LARC who received neoadjuvant CRT between 2016 and 2020 at FUSCC was selected for clinical validation (Fig. 1I). Kaplan-Meier survival analysis revealed that patients harboring the BRAF^V600E^ mutation had significantly shorter overall survival after receiving CRT compared to those with wild-type BRAF (HR: 8.19, 95% CI: 1.24–54.06, *p* < 0.0001) (Fig. 1J). Furthermore, the analysis of TRG distribution following neoadjuvant CRT also showed that BRAF^V600E^-mutant patients exhibited higher proportions of TRG2 (52.94% vs. 28.57%) and TRG3 (17.65% vs. 6.28%), and lower proportions of TRG0 (0% vs. 15.37%) and TRG1 (5.88% vs. 12.77%), indicating a poorer local tumor regression response to neoadjuvant CRT compared to wild-type patients (Fig. 1K).

Overall, the correlation analysis of driver gene mutation status and RT sensitivity in organoid models, combined with clinical data validation, preliminarily indicated that the BRAF^V600E^ mutation is associated with RT resistance in CRC.

### Identification and validation of BRAF^V600E^-mutant and wild-type organoids

**Fig. 2:**
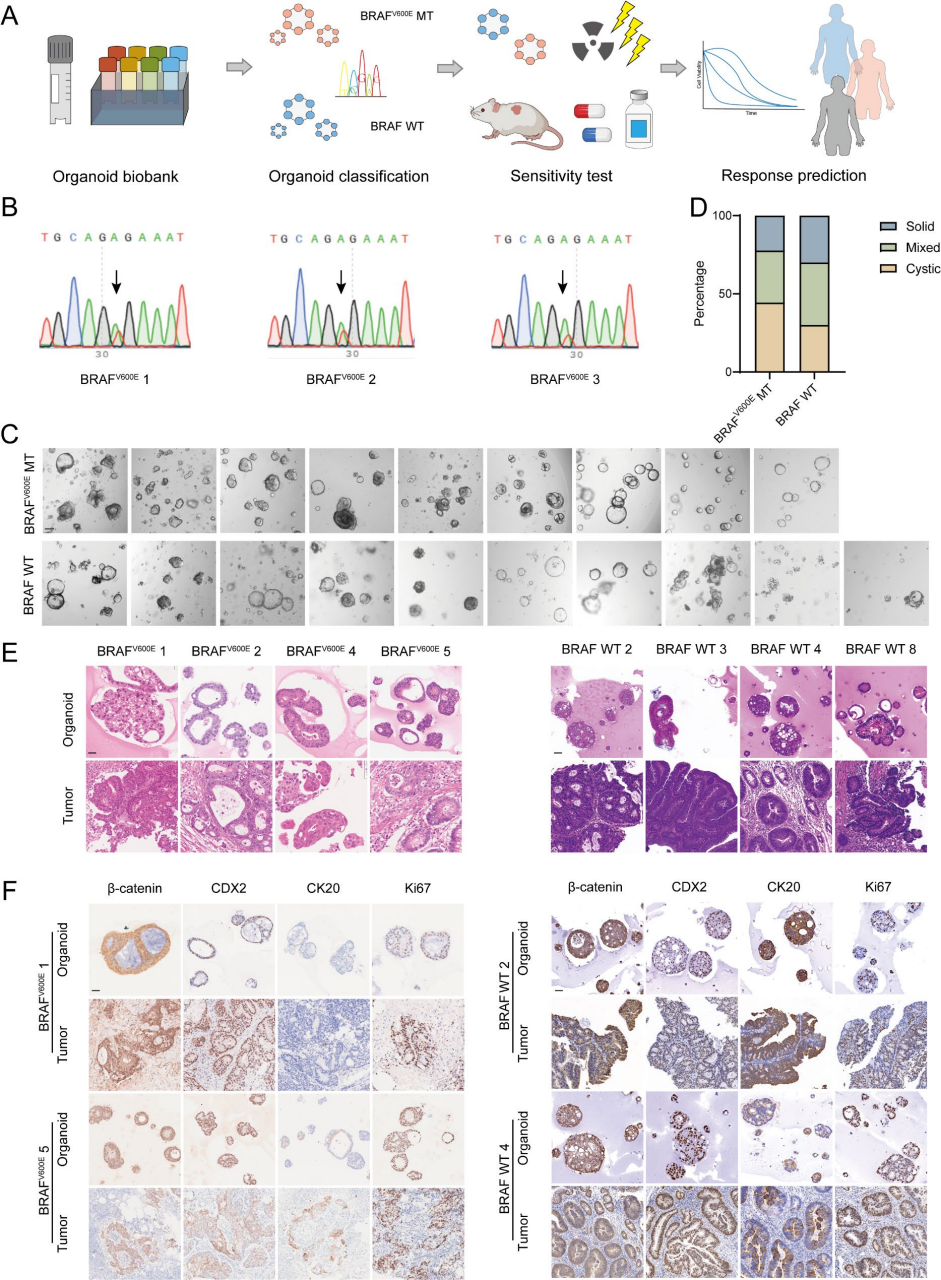
Identification and histopathological characterization of BRAF^V600E^ mutant and wild-type PDOs. (**A**) Graphical summary of the overall experimental design. An organoid biobank was constructed, and BRAF^V600E^ mutant and BRAF wild-type organoids were screened using Sanger sequencing. Drug sensitivity tests for radiation and chemotherapy were conducted both in vitro and in vivo, and the results were compared with clinical treatment outcomes for patients. (**B**) Sanger sequencing results of selected BRAF mutant organoids. The arrow indicated the BRAF^V600E^ mutation site. (**C**) The bright-field images representing growth morphology of BRAF^V600E^ mutant and BRAF wild-type organoids. Scale bar, 50 μm. (**D**) The morphology of BRAF^V600E^ mutant and BRAF wild-type organoids was classified into three typical categories. (**E**) H&E staining comparing patient derived organoids with corresponding primary tumors. Left: BRAF^V600E^ mutant organoids and tumors. Right: BRAF wild-type organoids and tumors. Scale bar, 20 μm. (**F**) Immunohistochemistry staining of β-catenin, CDX2, CK20, and Ki67 on tumor organoids and corresponding primary tumors. Left: BRAF^V600E^ mutant organoids and tumors. Right: BRAF wild-type organoids and tumors. Scale bar, 20 μm. See also Figure S1 and Table S2 (Supporting Information)

To further explore the relationship between radiosensitivity and BRAF mutations, we used the established CRC patient-derived organoid biobank for validation (Fig. 2A). Organoids were screened and selected using Sanger sequencing to identify those with the BRAF^V600E^ mutation and BRAF wild-type status (Fig. 2B, Figure S1), as the V600E mutation is the most common type of BRAF mutation [33]. Based on these sequencing results, 9 BRAF^V600E^-mutant organoids and 10 corresponding BRAF wild-type organoids were selected for further analysis and all 19 organoids were MSS (detailed patient information is available in Table S2).

Representative bright-field images of BRAF^V600E^-mutant and BRAF wild-type organoids demonstrated that organoids retained the morphological heterogeneity of tumors (Fig. 2C). Comparative analysis of the three typical morphological classifications between BRAF^V600E^-mutant and wild-type organoids revealed no significant differences in morphological distribution between the two groups (Fig. 2D). H&E staining indicated that organoids retained the histological characteristics of the original tumor tissues (Fig. 2E). Furthermore, the expression patterns of β-catenin, CDX2, CK20, and Ki67 were found to be similar between the organoids and tumor tissues, demonstrating that organoids preserve the molecular biological characteristics of the original tissues (Fig. 2F).

These results collectively demonstrated that 9 BRAF^V600E^-mutant and 10 BRAF wild-type CRC organoids were successfully identified and selected from the organoid biobank for subsequent experiments. The organoids preserved tumor heterogeneity and exhibited consistency with their source tissues, indicating that organoids can accurately reflect the characteristics of originating tumors.

### BRAF^V600E^-mutant organoids exhibit greater radioresistance compared to wild-type

Previous studies established a quantitative in vitro radiation sensitivity assay by irradiating mouse small and large intestinal organoids. The assay provided radiation profiles that mimicked the conditions of the original organs and was analyzed using the single hit multi-target (SHMT) algorithm [34]. Here, we adapted the principles of this system to evaluate the radiation sensitivity of BRAF^V600E^ mutated and wild-type organoids.

**Fig. 3:**
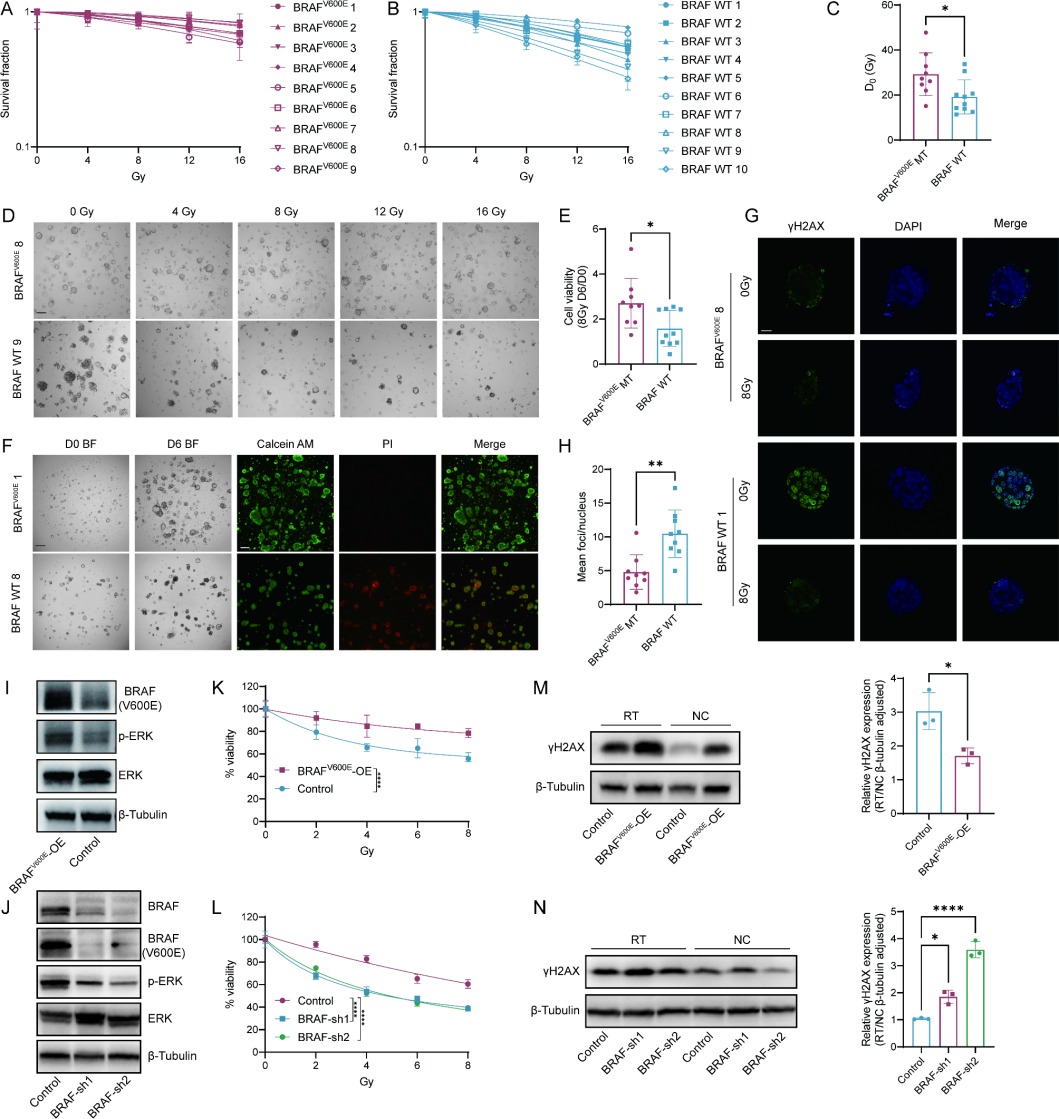
BRAF^V600E^ mutant organoids show higher resistance to radiation compared to wild-type organoids. (**A**) Dose survival curves of BRAF^V600E^ mutant PDOs irradiated with escalating doses (4–16 Gy). Data (mean ± SD) were collated from three different experiments. (**B**) Dose survival curves of BRAF wild-type PDOs irradiated with escalating doses (4–16 Gy). Data (mean ± SD) were collated from three different experiments. (**C**) Radiosensitivity (D_0_) of PDOs were compared between BRAF^V600E^ mutant and BRAF wild-type group. D_0_ was detected by the in vitro clonogenic radiation assay and statistical significance was analyzed by student’s t-test (29.27 vs. 19.21, *p* = 0.0199). Quantitation of D_0_ is presented as mean ± SD (*n* = 9 in BRAF^V600E^ MT group, *n* = 10 in BRAF WT group ). (**D**) Representative brightfield images of BRAF^V600E^ mutant PDO irradiated with escalating doses (top) compared with BRAF wild-type PDO (bottom). Images were captured 6 days post radiation. Scale bar, 100 μm. (**E**) Cell viability of PDOs were compared between BRAF^V600E^ mutant and BRAF wild-type group. Cell viability was measured 6 days after 8 Gy radiation and normalized to cell viability on day 0. Statistical significance was analyzed by student’s t-test (2.70 vs. 1.58, *p* = 0.0201). Quantitation of cell viability is presented as mean ± SD (*n* = 9 in BRAF^V600E^ MT group, *n* = 10 in BRAF WT group). (**F**) Representative images showing cell viability of BRAF^V600E^ mutant and wild-type organoids 6 days post 8 Gy radiation (Left: representative brightfield images. Scale bar, 100 μm. Right: representative fluorescent images stained with Calcein AM (green) for alive cells and PI (red) for dead cells. Scale bar, 200 μm). (**G**) Representative high-magnified immunofluorescent images of γH2AX foci (green) in one BRAF^V600E^ mutant PDO and BRAF wild-type PDO 10 h post 8 Gy radiation. Scale bar, 20 μm. Nuclei were stained by DAPI. (**H**) Quantitative analysis of focus resolution for γH2AX foci in BRAF^V600E^ mutant and BRAF wild-type organoids post 8 Gy radiation. Foci/ Nucleus were determined using the standard 8.28 pixel/ focus. Statistical significance was analyzed by student’s t-test (4.79 vs. 10.47, *p* = 0.0012). Quantitation of mean foci/nucleus is presented as mean ± SD (*n* = 9 per group). (**I**) BRAF ^V600E^ and p-ERK expression in Caco-2 cells transfected with BRAF ^V600E^ (BRAF^V600E^-OE) and blank vector (Control). (**J**) BRAF, BRAF^V600E^ and p-ERK expression in HT29 cells transfected BRAF-shRNA and blank vector (Control). **K**) Relative viability of Caco-2 cells transfected with BRAF V600E (BRAF^V600E^-OE) and blank vector (Control) exposed to increasing dosage of radiation (2–8 Gy). Statistical significance was analyzed by two-way ANOVA. Data represent mean ± SD (*n* = 4 per group). **L**) Relative viability of HT29 cells transfected with BRAF-shRNA and blank vector (Control) exposed to increasing dosage of radiation (2–8 Gy). Statistical significance was analyzed by two-way ANOVA. Data represent mean ± SD (*n* = 4 per group). **M**) γH2AX expression in BRAF^V600E^-OE and control Caco-2 cells before and after 4 Gy irradiation. **N**) γH2AX expression in BRAF-sh and control HT29 cells before and after 4 Gy irradiation

Radiation dose survival curves for 9 distinct BRAF^V600E^-mutant PDO lines exposed to increasing radiation doses showed a mean D_0_ (Gy) of 29.27 ± 9.49 (Fig. 3A). And the representative brightfield images demonstrated that the survival rate of BRAF^V600E^-mutant PDOs remained high even when exposed to doses as high as 16 Gy (Fig. 3D). However, survival curves for 10 BRAF wild-type organoids depicted higher levels of radiosensitivity following exposure to escalating doses at 6 days post-radiation, with a mean D_0_ (Gy) of 19.21 ± 7.56 (Fig. 3B). Representative bright-field images showed that a BRAF wild-type organoid exhibited noticeable cell death after exposure to 4 Gy radiation, with increased cell death becoming more apparent as the radiation dose increased (Fig. 3D). This phenomenon was more commonly observed in BRAF wild-type organoids and was less frequent in mutant types (Figure S2A, B). D₀ value of BRAF^V600E^ mutant organoids was significantly higher than that of wild-type organoids (29.27 vs. 19.21, *p* = 0.0199), demonstrating that BRAF^V600E−^mutant organoids exhibited greater radioresistance(Fig. 3C).

In addition to analyzing dose survival curves of PDOs irradiated with escalating doses, we also conducted an analysis of the organoid size ratio 24 days following exposure to 8 Gy radiation (Figure S3A, B). The size ratio in the BRAF^V600E^-mutant group was significantly higher than that in the wild-type group (329.9% vs173.4%, *p* = 0.0004) (Figure S3C). Representative bright-field images also indicated that BRAF mutant organoids exhibited larger sizes after 8 Gy irradiation for 24 days (Figure S3D). These findings once again underscored the radioresistant status of BRAF^V600E^-mutant organoids in response to RT.

Apart from exploring the radiosensitivity of organoids from a morphological perspective, we analyzed the changes in cell viability of organoids with different mutation status following radiation. Cell viability was measured 6 days after 8 Gy radiation using CellTiter-Glo (CTG) and normalized to cell viability on day 0. The results indicated that BRAF^V600E^-mutant organoids exhibited significantly higher cell viability 6 days post-irradiation (2.70 vs. 1.58, *p* = 0.0201) (Fig. 3E). Furthermore, live/dead cell staining showed, that BRAF^V600E^-mutant organoids not only had larger sizes compared to wild-type organoids post-irradiation but also exhibited a significantly higher proportion of live cells and a lower proportion of dead cells (Fig. 3F).

Radiation induces DNA damage primarily through the generation of DNA double-strand breaks (DSBs), which are critical lesions that can lead to cell death if not properly repaired. One of the earliest cellular responses to DSBs is the phosphorylation of the histone protein H2AX at serine 139, forming γH2AX. The level of γH2AX serves as a marker for DNA damage and repair efficiency, reflecting radiosensitivity [35, 36]. Here we detected γH2AX foci in PDOs according to the previously reported ionizing radiation–induced foci (IRIF) technology [26] and the number of γH2AX foci per nucleus was analyzed (Fig. 3G-H, Figure S2 C, D). Most BRAF^V600E^-mutant organoids exhibited fewer γH2AX foci per nucleus 10 h post-irradiation (4.79 ± 2.55), indicating that a considerable portion of DSBs had been repaired. However, a higher number of unresolved γH2AX IRIF still remained in BRAF wild-type organoids (10.47 ± 3.53). These findings suggested that BRAF^V600E^-mutant organoids possessed robust DNA damage repair capabilities, consistent with their observed radiation resistance in clonogenic assays.

Previous studies have shown that some organoids exhibit regrowth after treatment, correlating with treatment resistance and potentially linking to clinical drug resistance and tumor recurrence in patients [37, 38]. Therefore, we investigated the occurrence and frequency of regrowth in organoids with different mutation statuses (Figure S4A). The results revealed a significantly higher frequency of regrowth in BRAF^V600E^-mutant (5/ 9) compared to BRAF wild-type (1/ 10) organoids (Figure S4B). Brightfield images indicated that while BRAF^V600E^ mutants (e.g., BRAF^V600E^ 5 and BRAF^V600E^ 7) showed initial growth inhibition or death by day 6 post-irradiation, some exhibited regrowth by day 12. In contrast, most wild-type organoids, such as BRAF WT 1, did not show regrowth (Figure S4C). Representative immunofluorescence staining for Ki67 and CD44 demonstrated higher expression levels in regrown organoids post-irradiation, indicating enhanced proliferative capacity and stemness, suggesting that radiation resistance in BRAF^V600E^-mutant organoids might be related to these enhanced characteristics (Figure S4D).

To further validate the radiotherapy resistance of BRAF^V600E^-mutant CRC and enhance comparability, isogenic cell lines with BRAF^V600E^ mutation and wild-type were constructed. BRAF^V600E^ was overexpressed in the BRAF wild-type Caco-2 cells, while BRAF^V600E^ was knocked down in BRAF^V600E^-mutant HT29 cells, thereby inhibiting the hyperactivation of the MAPK signaling pathway caused by mutation (Fig. 3I, J). Radiotherapy sensitivity analysis showed that BRAF^V600E^-overexpressing cells exhibited significantly higher survival rates than wild-type cells after irradiation at different doses (Fig. 3K), with a smaller increase in γH2AX expression levels after radiation exposure (Fig. 3M). Conversely, in BRAF^V600E^-knockdown HT29 cells, cell viability decreased significantly after graded irradiation (Fig. 3L), and the change in γH2AX expression levels before and after radiation was more pronounced (Fig. 3N), further confirming the key role of BRAF mutation in radiotherapy resistance.

In addition to directly editing BRAF gene, we also inhibited BRAF activity using BRAF inhibitors to assess their impact on radiotherapy sensitivity. Based on previous study [39], IC70 concentrations were selected to balance drug and radiation efficacy (Figure S5A). Organoids were treated with radiation with or without BRAF inhibitors, and fitted to SHMT model. BRAF inhibition significantly reduced survival fractions, with sensitizer enhancement ratios at 10% survival (SER10%) exceeding 1, confirming a radiosensitizing effect (Figure S5B-E). Cell viability assays six days post 8 Gy irradiation further supported this synergy, with combination treatment reducing viability and organoid size more effectively than monotherapy (Figure S5F-J). Similarly, in HT29 cells, clonogenic assays demonstrated that Vemurafenib significantly reduced colony formation ability, with an SER10% of 1.20 (Figure S5K, L), while cell viability also showed a marked decrease following combination treatment (Figure S5M, N). Consistently, γH2AX expression was elevated in BRAF^V600E^-mutant cell lines and organoids upon combined radiotherapy and BRAF inhibitor treatment (Figure S5O-Q).

Taken together, these results systematically described the radiobiological properties of BRAF^V600E^-mutant CRC and highlighted its radioresistance.

### BRAF^V600E^-mutant organoids demonstrate chemotherapy resistance and reflect clinical outcome

**Fig. 4:**
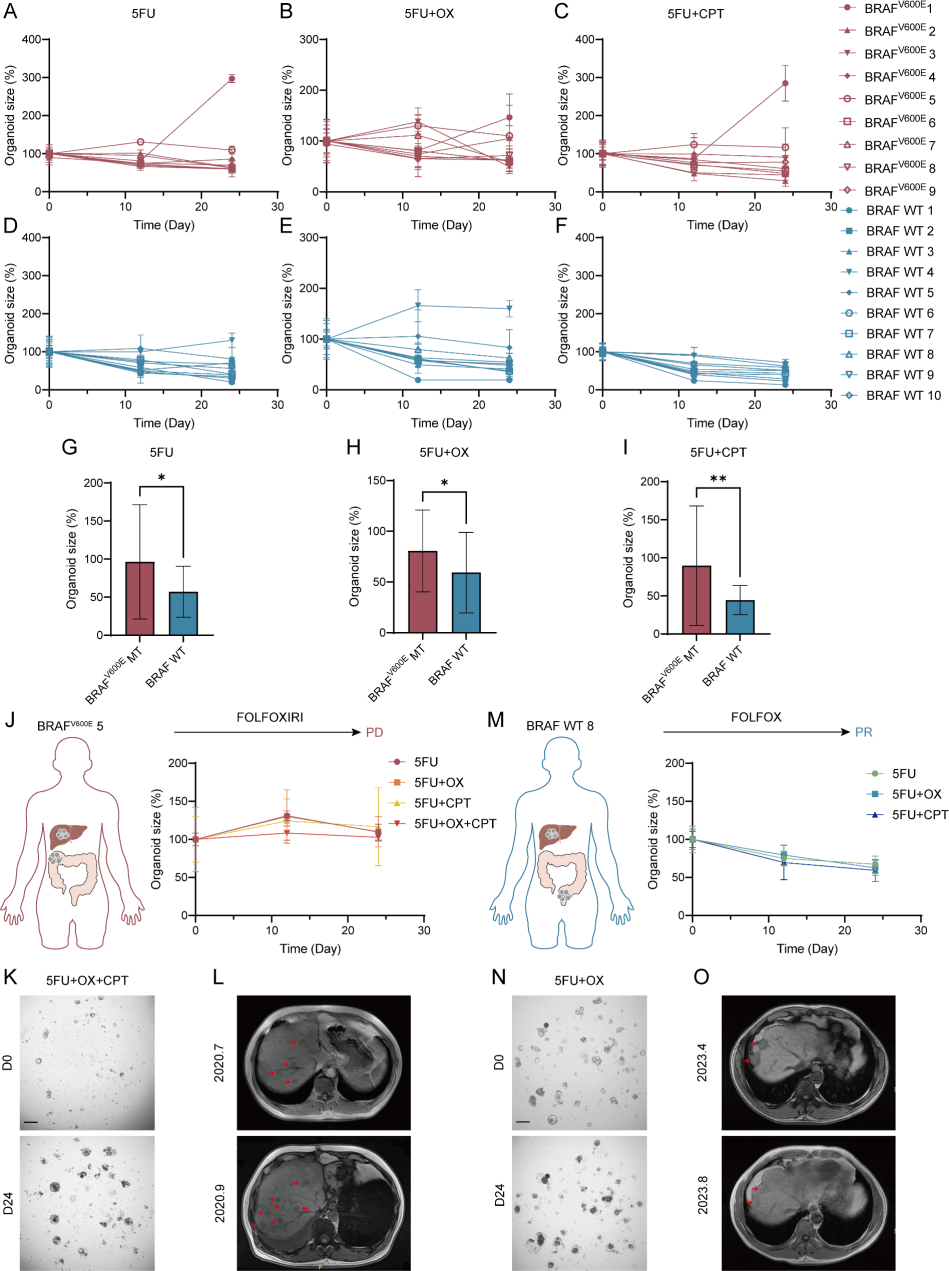
BRAF^V600E^ mutant organoids are resistant to chemotherapy compared to wild-types and can reflect clinical treatment outcomes in patients. **A**-**C**) Size changes in BRAF^V600E^ mutant organoids after different chemotherapy regimens (Left: 5FU. Middle: 5FU + OX. Right: 5FU + CPT). Data (mean ± SD) were collated from three different experiments. **D-F**) Size changes in BRAF wild-type organoids after different chemotherapy regimens (Left: 5FU. Middle: 5FU + OX. Right: 5FU + CPT). Data (mean ± SD) were collated from three different experiments. **G-I**) The ratio of organoid size at day 24 to the size at day 0 after different chemotherapy regimens in BRAF^V600E^ mutant and BRAF wild-type organoids (Left: 5FU, middle: 5FU + OX, right: 5FU + CPT). Statistical significance was analyzed by student’s t-test (5FU: 96.41% vs. 57.17%, *p* = 0.0121; 5FU + OX: 80.65% vs. 59.26%, *p* = 0.0488; 5FU + CPT: 89.70% vs. 44.45%, *p* = 0.0034). Quantitation of organoid size ratio is presented as mean ± SD (*n* = 9 in BRAF^V600E^ MT group, *n* = 10 in BRAF WT group, each organoid with three different experiments). **J-L**) Clinical treatment response of BRAF^V600E^ 5 patient and drug sensitivity tests of corresponding organoid. J: Organoid size changes after different chemotherapies. Data (mean ± SD) were collated from three different experiments. The size of organoids did not decrease under four different drug regimens, consistent with the clinical evaluation of PD in the patient after receiving FOLFOXIRI treatment. K: Representative bright-field images of BRAF^V600E^ 5 organoid on day 0 and day 24 after treatment with 5FU + OX + CPT. Scale bar, 100 μm. L: The imaging manifestations of target lesions in BRAF^V600E^ 5 patient before and after treatment. Arrows indicated intrahepatic tumor lesions. **M-O**) Clinical treatment response of BRAF WT 8 patient and drug sensitivity tests of corresponding organoid. M: Organoid size changes after different chemotherapies. Data (mean ± SD) were collated from three different experiments. The size of organoids decreased significantly under three different drug regimens, consistent with the clinical evaluation of PR in the patient after receiving FOLFOX treatment. N: Representative bright-field images of BRAF WT 8 organoid on day 0 and day 24 after treatment with 5FU + OX. Scale bar, 100 μm. O: The imaging manifestations of target lesions in BRAF WT 8 patient before and after treatment. Arrows indicated intrahepatic tumor lesions

Apart from RT, chemotherapy plays a foundational role in the treatment of CRC. Previous clinical trials have demonstrated that BRAF^V600E^-mutant CRC exhibit high malignancy and resistance to conventional 5FU-based chemotherapy regimens [40–42]. Thus, to investigate the chemosensitivity of BRAF^V600E^-mutant CRC, we used organoid models and validated the consistency between organoid experimental results and clinical outcomes. Organoid size changes were measured after treatment with different chemotherapy regimens: 5FU, 5FU + OX, and 5FU + CPT for 24 days (Fig. 4A-C for BRAF^V600E^ mutants and 4D-F for wild-types). After 24 days of chemotherapy, the size ratio of BRAF^V600E^ mutant organoids was significantly higher than that of wild-type organoids (Fig. 4G-I, 5FU: 96.41% vs. 57.17%, *p* = 0.0121; 5FU + OX: 80.65% vs. 59.26%, *p* = 0.0488; 5FU + CPT: 89.70% vs. 44.45%, *p* = 0.0034), indicating greater chemoresistance in the mutants.

Furthermore, 5-fluorouracil (5FU) dose-response assays showed that BRAF^V600E^ overexpression in Caco-2 cells increased 5FU resistance, with an IC50 of 26.48 μM compared to 7.29 μM in wild-type cells (Figure S6A). Colony formation assays further confirmed this resistance (Figure S6B). Similarly in HT29 cells, BRAF knockdown reduced 5FU resistance, lowering IC50 values from 3.02 μM in wild-type HT29 to 1.47 μM and 0.76 μM in BRAF-sh1 and BRAF-sh2 cells, respectively (Figure S6C).

Combination treatment with 5FU and BRAF inhibitors in HT29 cells showed synergistic cytotoxicity across various doses, as indicated by the Combination Index (CI) (Figure S6D, E). This synergy was also observed in BRAF^V600E^-mutant organoids, where 5FU and Vemurafenib co-treatment significantly reduced organoid viability and size (Figure S6F–H). These findings highlight BRAF’s role in chemoresistance.

To further investigate the correlation between organoid drug sensitivity assays and patient clinical outcomes, one BRAF^V600E^-mutant and one wild-type organoid were selected. Both organoids originated from CRC patients with liver metastasis, sharing the same TNM stage (T3N2M1) and histological type (adenocarcinoma). The BRAF^V600E^ 5 organoid did not reduce in size under four different chemotherapy regimens, indicating treatment resistance. Clinically, this patient showed progressive disease (PD) after FOLFOXIRI treatment, with increased and enlarged liver metastases (Fig. 4J-L). Conversely, the BRAF WT 8 organoid showed significant size reduction under three chemotherapy regimens, consistent with a partial response (PR) after FOLFOX treatment in the corresponding patient (Fig. 4M-O). These results collectively demonstrated that BRAF^V600E^-mutant organoids exhibited chemotherapy resistance compared to wild-type organoids, consistent with previous clinical trials. Moreover, the in vitro organoid drug sensitivity tests could faithfully reflect patient clinical outcomes, highlighting the potential of organoids for predicting treatment efficacy and guiding personalized therapy.

### Enhanced therapeutic efficacy of combined chemoradiotherapy in BRAF^V600E^-mutant organoids associated with increased early apoptosis

The combination of radiotherapy and chemotherapy is pivotal in CRC treatment, significantly enhancing tumor control and leading to improved patient outcomes. Chemoradiotherapy is particularly critical in managing LARC, often leading to tumor downstaging, which facilitates surgical resection and reduces recurrence and metastasis [43, 44]. However complete tumor response is observed only in 20–30% patients [45], making it crucial to identify those who can benefit from chemoradiotherapy.

**Fig. 5:**
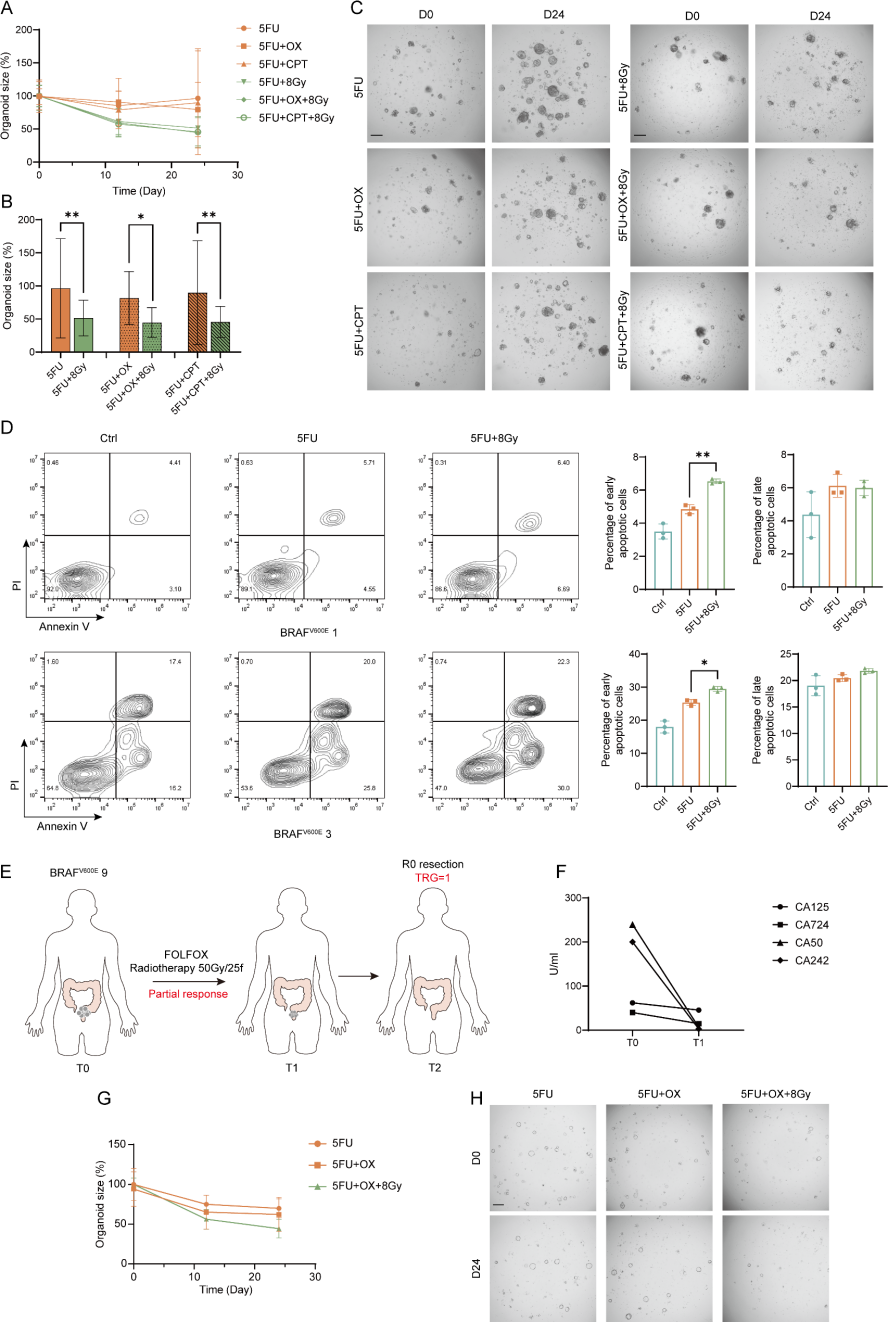
Chemoradiotherapy is more effective than chemotherapy alone in BRAF^V600E^ mutant organoids and is associated with increased early apoptosis. (**A**) Size changes in BRAF^V600E^ mutant organoids after 5FU, 5FU + OX, 5FU + CPT treatment and each combined with 8 Gy radiation. Data (mean ± SD) were collated from 9 organoids and each with three different experiments. (**B**) The ratio of organoid size at day 24 to the size at day 0 after 5FU, 5FU + OX, 5FU + CPT treatment and each combined with 8 Gy radiation. Statistical significance was analyzed by one-way ANOVA (5FU vs. 5FU + 8 Gy: 96.41% vs. 51.49%, *p* = 0.0079; 5FU + OX vs. 5FU + OX + 8 Gy: 81.76% vs. 44.63%, *p* = 0.0492; 5FU + CPT vs. 5FU + CPT + 8 Gy: 89.70% vs. 45.54%, *p* = 0.0096 ). Quantitation of organoid size ratio is presented as mean ± SD (9 organoids per group and each organoid with three different experiments). (**C**) Representative bright-field images of BRAF^V600E^ 1 organoid on day 0 and day 24 after treatment with 5FU, 5FU + OX, 5FU + CPT and each combined with 8 Gy radiation. Scale bar, 100 μm. (**D**) Quantification of apoptotic subsets (Early apoptotic: Annexin V + PI-, late apoptotic and necrosis: Annexin V + PI+) within total cells of BRAF^V600E^ mutant organoids by flow cytometry (Top: BRAF^V600E^ 1, bottom: BRAF^V600E^ 3). Statistical significance was analyzed by one-way ANOVA BRAF^V600E^ 1: 6.53% vs. 4.84%, *p* = 0.0015; BRAF^V600E^ 3: 29.47% vs. 25.37%, *p* = 0.0164 ). Data (mean ± SD) were collated from three different experiments. (**E**) Clinical treatment course and efficacy evaluation of BRAF^V600E^ 9 patient. (**F**) The comparison of tumor markers of BRAF^V600E^ 9 patient before and after receiving neoadjuvant chemoradiotherapy. (**G**) Size changes in BRAF^V600E^ 9 organoid after 5FU, 5FU + OX and 5FU + OX + 8 Gy. Data (mean ± SD) were collated from three different experiments. (**H**) Representative bright-field images of BRAF^V600E^ 9 organoid on day 0 and day 24 after treatment with 5FU, 5FU + OX and 5FU + OX + 8 Gy. Scale bar, 100 μm

So as to investigate the sensitivity of BRAF^V600E^-mutant CRC to combined chemoradiotherapy, we treated 9 BRAF^V600E^-mutant organoids with 5FU, 5FU + OX, and 5FU + CPT chemotherapy regimens, each combined with 8 Gy RT. (Fig. 5A). Results indicated that while BRAF^V600E^-mutant organoids were resistant to chemotherapy or RT alone, they were relatively sensitive to the combination, showing significantly reduced size in the chemoradiotherapy treatment group (5FU vs. 5FU + 8 Gy: 96.41% vs. 51.49%, *p* = 0.0079; 5FU + OX vs. 5FU + OX + 8 Gy: 81.76% vs. 44.63%, *p* = 0.0492; 5FU + CPT vs. 5FU + CPT + 8 Gy: 89.70% vs. 45.54%, *p* = 0.0096) (Fig. 5B). Representative brightfield images also confirmed these findings (Fig. 5C). Further investigation into the mechanism of the combined treatment using two representative organoids revealed that early apoptosis significantly increased in the combined group (BRAF^V600E^ 1: 6.53% vs. 4.84%, *p* = 0.0015; BRAF^V600E^ 3: 29.47% vs. 25.37%, *p* = 0.0164), whereas late apoptosis and necrosis did not show significant changes (Fig. 5D). This suggested that the synergistic effect of chemoradiotherapy in BRAF^V600E^-mutant organoids might be related to the promotion of early apoptosis.

In the case of BRAF^V600E^ 9 patient, who received FOLFOX chemotherapy combined with 50 Gy/25 fractions of RT, the treatment evaluation showed a partial response (PR), with a significant decrease in tumor marker levels post-treatment (Fig. 5F). The patient achieved an R0 resection after neoadjuvant chemoradiotherapy, and the postoperative pathology showed TRG = 1, indicating a relatively good response to the neoadjuvant chemoradiotherapy (Fig. 5E). Correspondingly, the organoids from this patient showed a significant reduction in size after receiving 5FU combined with OX and 8 Gy RT compared to chemotherapy alone, consistent with the clinical treatment outcome (Fig. 5G-H). These results together underscored the importance and necessity of combined chemoradiotherapy in patients with BRAF^V600E^ mutations.

### Chemoradiotherapy collaboratively inhibit tumor progression in BRAF^V600E^-mutant patient-derived organoid xenograft (PDOX) mouse models

To validate our in vitro experimental results, we subcutaneously implanted BRAF^V600E^ 1 organoids in two 6-week-old female NSG mice. Upon the tumors reaching 500 mm³, they were dissected and re-transplanted into female BALB/c nude mice. The histological consistency between PDOX derived from nude mice, organoids, and patient tumor tissues was confirmed using H&E staining (Figure S7A).

**Fig. 6:**
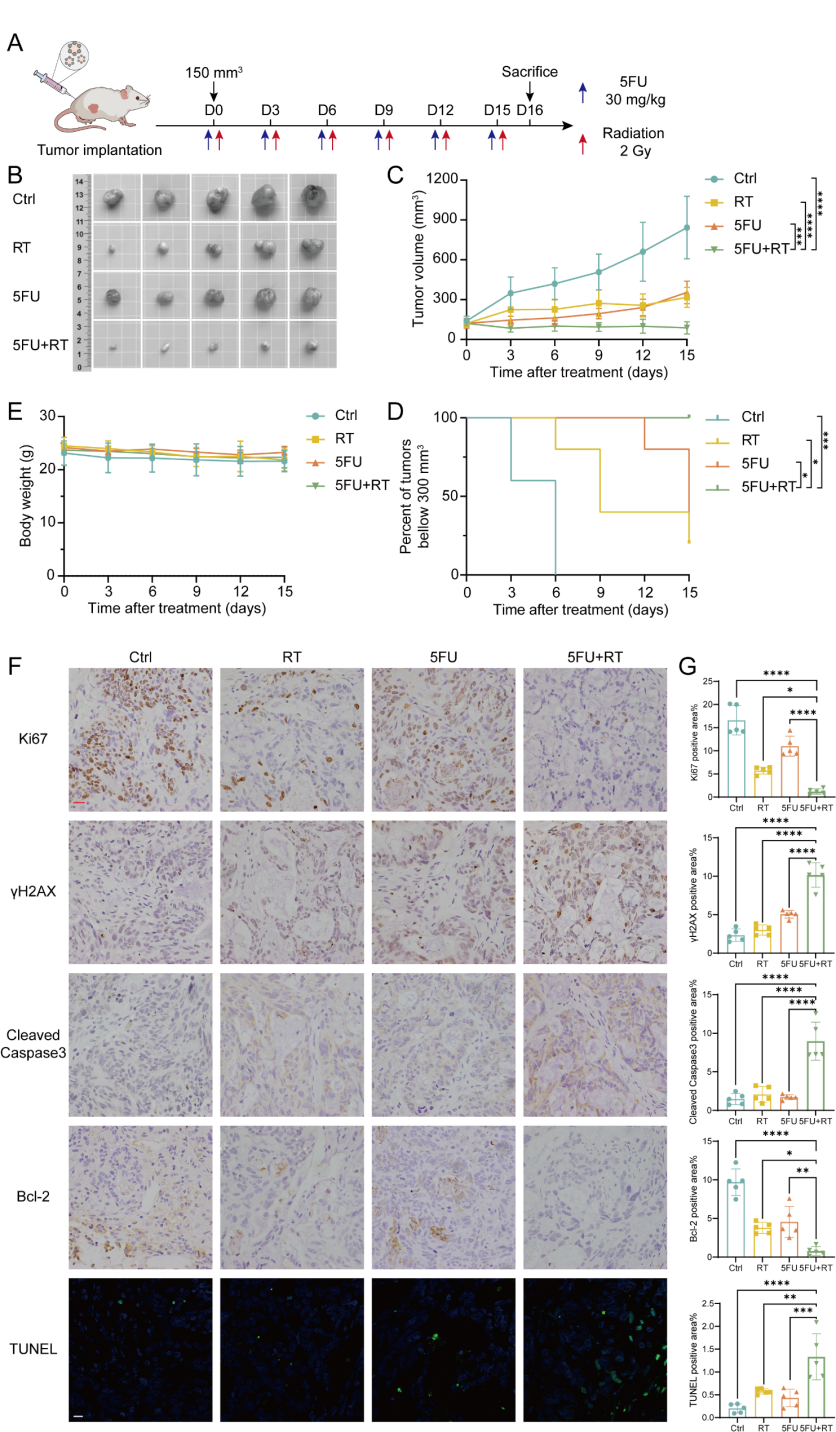
Combined chemoradiotherapy exhibits stronger tumor suppression compared to either radiotherapy or chemotherapy alone in PDOX mouse models. (**A**) Diagram of experimental protocol for in vivo treatment of BRAF^V600E^ PDOX mouse models. BRAF^V600E^ 1 organoids were implanted into flanks of BALB/c nude mice (previously passaged in NSG mouse), and once upon tumors reaching an average size of 100–150 mm³, mice were assigned randomly to the following treatment groups: Ctrl (PBS + untreated), RT (PBS + 2 Gy radiation), 5FU (5FU 30 mg/kg + untreated), or 5FU + RT (5FU 30 mg/kg + 2 Gy radiation). Both 5FU and 2 Gy radiation were administered on the same day, with treatments occurring every three days. Mouse were sacrificed 16 days post the start of treatment for further analysis. (**B**) Tumors from different treatment groups were imaged after isolation from mouse flanks on day 16. (**C**) Tumor volumes for the indicated treatment from day 0 to 15. Statistical significance was analyzed by two-way ANOVA (5FU + RT vs. 5FU: 97.99 vs. 203.3, *p* = 0.0003; 5FU + RT vs. RT: 97.99 vs. 236.2, *p* < 0.0001; 5FU + RT vs. Ctrl: 97.99 vs. 486.3, *p* < 0.0001). Data represent mean tumor volumes ± SD (*n* = 5 per group). (**D**) Kaplan–Meier analysis showed the overall survival of BRAF^V600E^ organoid bearing mice in different treatment groups and analyzed by log-rank test (5FU + RT vs. 5FU: *p* = 0.0158, 5FU + RT vs. RT: *p* = 0.0135, 5FU + RT vs. Ctrl: *p* = 0.0031) (*n* = 5 per group). (**E**) Body weight for the indicated treatment from day 0 to 15. Data represent mean body weights ± SD (*n* = 5 per group). (**F**) Representative immunohistochemistry staining images of Ki67, Bcl-2, Cleaved Caspase-3 and γH2AX (Scale bar, 20 μm) and TUNEL staining images in different treatment groups (Scale bar,10 μm). (**G**) The percentage of positive areas for Ki67, Bcl-2, Cleaved Caspase-3, and γH2AX immunohistochemical staining, as well as TUNEL staining, was compared among different treatment groups. Statistical significance was analyzed by one-way ANOVA. Quantitation of percentage of positive area is presented as mean ± SD (*n* = 5 per group)

Once tumors reached an average volume of 100 ∼ 150 mm^3^, mice were randomized into the following treatment groups, with 5 mice in each group: Ctrl (PBS + untreated), RT (PBS + 2 Gy radiation), 5FU (5FU 30 mg/kg + untreated) or 5FU + RT (5FU 30 mg/kg + 2 Gy radiation). 5FU and 2 Gy radiation were administered on the same day, with treatment given every 3 days (Fig. 6A).

Consistent with the in vitro organoid experiments, both the 5FU and RT groups significantly inhibited tumor growth compared to the Ctrl group. However, the 5FU + RT group exhibited the most substantial tumor suppression, indicated by the smallest tumor volume and significantly prolonged overall survival (Fig. 6B-D). Additionally, the combined treatment group showed no notable increase in treatment-related adverse effects, as evidenced by stable body weights across the four groups (Fig. 6E), no significant main organ toxicity (Figure S7E), and normal blood and liver/kidney function tests (Figure S7B-D). Immunohistochemical staining of tumor tissues revealed that the combination of 5FU + RT synergistically suppressed the expression of proliferation marker Ki67 and anti-apoptotic protein Bcl-2 while promoting the expression of apoptotic marker Cleaved Caspase-3 and DNA damage marker γH2AX, consistent with TUNEL staining results (Fig. 6F, G). These findings collectively indicated that in BRAF^V600E^-mutant PDOX mouse models, the combination of RT and chemotherapy exhibited superior tumor suppression compared to either treatment alone, promoting tumor apoptosis and inhibiting proliferation, thereby suggesting that CRC patients with BRAF^V600E^ mutations might benefit from combined chemoradiotherapy.

## Discussion

RT is a fundamental component in the therapeutic arsenal for CRC, particularly in the neoadjuvant CRT setting for rectal cancer [3]. However, the efficacy of RT is not uniformly experienced across patients, prompting an exploration into the genetic underpinnings that may dictate radiosensitivity. Previous studies have shown that mutations in KRAS and TP53 are associated with CRT sensitivity [10, 12, 13]. Our study leverages PDOs to dissect the intricate relationship between genetic mutations, specifically BRAF^V600E^, and the radiobiological response. Although some studies have explored CRT sensitivity in BRAF-mutant CRCs [5, 14, 19], their conclusions are inconsistent, with small sample sizes and lack of validation, highlighting the need for further investigation into the radiobiological characteristics of this kind of tumor.

In this study, we focused on MSS-type colorectal cancer with the BRAF^V600E^ mutation. Although BRAF^V600E^ mutations are highly correlated with deficient mismatch repair (dMMR) protein expression and microsatellite instability-high (MSI-H) disease, MSI-H CRC is predominantly treated with immunotherapy, with unfavorable efficacy of chemoradiotherapy. Moreover, previous studies have shown that BRAF mutation status does not affect the immune checkpoint inhibitor response [33, 46–48]. Therefore, investigating the role of radiotherapy and chemoradiotherapy in MSS-type CRC holds greater clinical relevance.

The comprehensive analysis presented in this study, encompassing organoid survival curves, morphological assessments, cell viability measurements, and DNA damage response profiling, offers a multifaceted perspective on the radiobiological behavior of BRAF-mutant CRC. This systematic characterization is the first of its kind, extensively detailing the radiobiological phenotype. Our findings not only contribute to the molecular oncology of CRC but also hold significant potential for the development of personalized treatment strategies, emphasizing the integral role of RT in the comprehensive management of BRAF-mutant CRC.

The validation of our in vitro PDO findings with clinical outcomes underscores the predictive power of these models in anticipating patient response to therapy. This concordance not only bolsters the credibility of our research but also paves the way for the clinical application of PDOs in tailoring RT regimens to individual genetic profiles. In addition to radiosensitivity, we also explored the sensitivity of BRAF^V600E^-mutant organoids to chemotherapy and combined CRT, with validation in PDOX mouse models. Results showed that while BRAF^V600E^-mutant organoids were resistant to either RT or chemotherapy alone, they exhibited relatively better responses to CRT, highlighting the importance of RT in combined treatments and offering valuable insights for clinical decision-making. Deborah Schrag et al. performed a randomized phase III trial to compare FOLFOX chemotherapy with selective use of 5FUCRT (intervention) to 5FUCRT (control) for neoadjuvant treatment for LARC and found that FOLFOX chemotherapy with selective use of 5FUCRT is non-inferior to 5FUCRT [49]. However, the genetic characteristics of patients suitable for treatment regimens with RT-free remain unclear. Our findings suggested that BRAF^V600E^-mutant LARC patients may not be the candidate for RT-free protocols.

Although CRT showed better efficacy than RT or chemotherapy alone in organoid studies, BRAF-mutant patients in our clinical cohort of LARC receiving neoadjuvant CRT still had poorer outcomes compared to non-mutant patients. This aligns with previous research [14], indicating that CRT alone may not be sufficient enough for BRAF-mutant CRC patients. Therefore, new combined treatment strategies, such as BRAF-targeted therapy and immunotherapy, need to be explored. Considering RT’s potential role in combination treatments, it should be included in the exploration of comprehensive therapeutic approaches. Indeed, Ryan Robb et al. found that BRAF inhibitor selectively radiosensitized BRAF^V600E^ thyroid cancer cells through inhibiting NHEJ [50] and Tina Dasgupta et al. demonstrated RT + PLX4720 exhibited greater anti-tumor effects than either monotherapy in BRAF^V600E^ high-grade gliomas [51], suggesting that these combined approaches hold promise and warrants further investigation.

Although this study held clinical significance, it had some limitations. Due to the rarity of BRAF^V600E^-mutant CRC in clinical patients, the number of organoid samples used in this research was limited. This may restrict the generalizability of the results, somewhat affecting their clinical translational value. More replication in further studies is necessary before these findings can be applied clinically.

## Conclusions

This study provides a robust analysis of the correlation between RT sensitivity and genetic mutations in CRC, with a particular focus on BRAF^V600E^ mutations. The extensive in vitro and in vivo experiments revealed the radiobiological characteristics of BRAF^V600E^-mutant CRC, demonstrating its resistance to radiotherapy, while highlighting the role of radiotherapy in combined chemoradiotherapy. The consistency between the organoid experiment results and clinical outcomes in patients offers new insights for the treatment of this subset of patients.

## Electronic supplementary material

Below is the link to the electronic supplementary material.

Supplementary Material 1

Supplementary Material 2

Supplementary Material 3

## Data Availability

The data utilized in this study can be obtained from the corresponding author upon reasonable request.

## Abbreviations

5FU: 5-Fluorouracil
AD: Adenocarcinomas
CI: Combination Index
CPT: Irinotecan
CRC: Colorectal cancer
CRT: Chemoradiotherapy
DFS: Disease-free survival
dMMR: Deficient mismatch repair
DSBs: Double-strand breaks
LACR: Locally advanced rectal cancer
MC: Mucinous adenocarcinoma
MSI-H: Microsatellite instability-high
MSS: Microsatellite stable
NGS: Next-generation sequencing
OX: Oxaliplatin
PD: Progressive disease
PDOs: Patient-derived organoids
PR: Partial response
RT: Radiotherapy
SER10%: Sensitizer enhancement ratios at 10% survival
SHMT: Single hit multi-target
WES: Whole-exome sequencing
