# Supplementary material for: Unveiling radiobiological traits and therapeutic responses of BRAFV600E-mutant colorectal cancer via patient-derived organoids
Source: J Exp Clin Cancer Res. 2025 Mar 11;44:92. doi: 10.1186/s13046-025-03349-z (PMC11895145; doi:10.1186/s13046-025-03349-z)
Supplement: Supplementary file 1 — Supplementary Material 1 [file 13046_2025_3349_MOESM1_ESM.docx]

**Supporting Information**

This Supporting Information file includes:

**(1)** **Supplementary Methods**

**(2) Supplementary Figures**

**Fig. S1.** Sanger sequencing results of CRC PDOs, related to Figure 2.

**Fig. S2.** Representative brightfield images of organoids irradiated with escalating doses and immunofluorescent images of γH2AX foci 10 hours after 8 Gy radiation, related to Figure 3.

**Fig. S3.** Comparison of the size ratio 24 days after 8 Gy radiation between BRAF^V600E^-mutant and wild-type organoids.

**Fig. S4.** Comparison of regrowth between BRAF^V600E^-mutant and wild-type organoids after high-dose radiation.

**Fig S5.** Radiosensitizing effects of BRAF inhibitors in BRAFV600E mutant CRC.

**Fig S6.** Impact of BRAF^V600E^ mutation on 5FU resistance and synergistic effect with BRAF Inhibitors.

**Fig. S7.** Comparison of histological consistency between PDOX tissue and the original tissue and the toxicity responses in different treatment groups, related to Figure 6.

**Supplementary Methods**

**KEY RESOURCES TABLE**

| **REAGENT or RESOURCE** | **SOURCE** | **IDENTIFIER** |
| --- | --- | --- |
| **Antibodies** | | |
| Mouse monoclonal  anti-Ki-67 | Cell Signalling  Technology | Cat# 9449  RRID: AB_2797703 |
| Rabbit monoclonal  anti-CDX2 | Cell Signalling  Technology | Cat# 12306  RRID: AB_2797879 |
| Rabbit monoclonal  anti-β-Catenin | Cell Signalling  Technology | Cat# 8480  RRID: AB_11127855 |
| Rabbit monoclonal  anti-Cytokeratin 20 | Cell Signalling  Technology | Cat# 13063  RRID: AB_2798106 |
| Mouse monoclonal  anti-Bcl-2 | Cell Signalling  Technology | Cat# 15071  RRID: AB_2744528 |
| Rabbit monoclonal  anti-Cleaved Caspase-3 | Cell Signalling  Technology | Cat# 9664  RRID: AB_2070042 |
| Rabblt monoclonal  anti-Phospho-H2AX-S139 | Abclonal | Cat# AP0687  RRID: AB_2863808 |
| Rabbit polyclonal  anti-CD44 | Proteintech | Cat# 15675-1-AP  RRID: AB_2076198 |
| Rabbit monoclonal  anti-BRAF(V600E) | Invitrogen | Cat# MA5-24661  RRID: AB_2661889 |
| Mouse monoclonal  anti-BRAF | Santa Cruz | Cat# sc-5284 RRID: AB_626760 |
| Rabbit polyclonal  anti-phospho-ERK1/2 | Abclonal | Cat# AP0472  RRID: AB_2756833 |
| Rabbit polyclonal  anti-ERK1/2 | Proteintech | Cat# 11257-1-AP  RRID: AB_2139822 |
| Rabbit polyclonal  anti-Beta Tubulin | Proteintech | Cat# 10094-1-AP  RRID: AB_2210695 |
| **Biological Samples** | | |
| Biopsy tissues of CRC patients | This study | N/A |
| **Chemicals, Peptides, and Recombinant Proteins** | | |
| Advanced DMEM/F12 | Gibco | Cat# 12634-010 |
| HEPES | Gibco | Cat# 15630080 |
| GlutMAX | Gibco | Cat# 35050-061 |
| Penicillin/streptomycin | Solarbio | Cat# P1400 |
| Sodium pyruvate | Gibco | Cat# 11360070 |
| NEAA | Gibco | Cat# 11140050 |
| Polybrene | Yeasen | Cat# 40804ES76 |
| Puromycin | Yeasen | Cat# 54752ES08 |
| N2 | Gibco | Cat# 17502-048 |
| B27 | Gibco | Cat# 17504-044 |
| EGF | Sino Biological | Cat# 50482-MNCH |
| N-Acetyl-L-cysteine | Sigma-aldrich | Cat# A9165 |
| Nicotinamide | Sigma-aldrich | Cat# N0636 |
| Normocin | invivogen | Cat# ant-nr-2 |
| Gentamicin/AmphoteritinB | Gibco | Cat# R01510 |
| A83-01 | Tocris | Cat# 2939 |
| Prostaglandin E2 | Sigma-aldrich | Cat# P6532 |
| Gastrin | Sigma-aldrich | Cat# G9145 |
| SB202190 | Sigma-aldrich | Cat# S7067 |
| R-spondin-1 | Sino Biological | Cat# 11083-HNAS |
| Noggin | Sino Biological | Cat# 50688-M02H |
| DMEM medium | Hyclone GE Healthcare | Cat# SH30243.01 |
| NGC organoid anti-adherence solution | D1Med | Cat# D23025-0050 |
| Bovine serum albumin | BBILife Science | Cat# A600332-0100 |
| Y-27632 dihydrochloride | Sigma-aldrich | Cat# Y0503 |
| Matrigel | Corning | Cat# 356231 |
| TrypLETM Express | GIBCO | Cat#12605-010 |
| CELLBANKERTM 2 | ZENOAQ | Cat#170905 |
| Citrate antigen retrieval  solution | Servicebio | Cat# G1202 |
| Donkey serum | Solarbio | Cat# SL050 |
| 5-Fluorouracil | Selleck | Cat# S1209 |
| Irinotecan | Selleck | Cat# S2217 |
| Oxaliplatin | Selleck | Cat# S1224 |
| Vemurafenib | MCE | Cat# HY-12057 |
| Dabradenib | Selleck | Cat# S2807 |
| Phosphate buffered saline | BasalMedia | Cat# B320KJ |
| Fetal bovine serum | GIBCO | Cat# 10270 |
| Hematoxylin solution | Servicebio | Cat# G1005-1 |
| Eosin solution | Servicebio | Cat# G1005-2 |
| 2×Hieff® PCR Master Mix | YEASEN | Cat# 10102 |
| Calcein AM | YEASEN | Cat# 40719ES50 |
| Propidium Iodide | YEASEN | Cat# 40755ES64 |
| Cell counting kit-8 | GlpBio | Cat# GK100001 |
| ProLong Diamond Antifade  Mountant with DAPI | ThermoFisher | Cat# P36971 |
| Bluing Solution | Servicebio | Cat# G1040 |
| Hydrochloric acid alcohol | Servicebio | Cat# G1039 |
| **Critical Commercial Assays** | | |
| TIANamp Genomic DNA Kit | TIANGEN | Cat# DP304-02 |
| One-step TUNEL kit | Servicebio | Cat# G1504 |
| PE Annexin V /propidium iodide  apoptosis detection kit | BD Pharmingen | Cat# 559763 |
| Immunohistochemistry kit  (anti-rabbit secondary antibody) | Servicebio | Cat# G1215 |
| Immunohistochemistry kit  (anti-mouse secondary antibody) | Servicebio | Cat# G1216 |
| CellTiter-Glo 3D Cell viability assay | Promega | Cat# G9683 |
| **Experimental Models: Cell Lines** | | |
| Human: CRC organoids | This study | N/A |
| **Software and Algorithms** | | |
| R software | GNU project | https://www.r-project. org/ |
| Image-Pro Plus 6.0 | Media Cybernetics | https://mediacy.com /image-pro/ |
| ImageJ | ImageJ Wiki | https://imagej.net/ |
| FlowJo version 10 | FlowJo | https://www.flowjo.com/ |
| GraphPad Prism 9 | GraphPad | [https://www.graphpad.com/ scientific-software/prism/](https://www.graphpad.com/scientific-software/prism/) |
| **Other** | | |
| Genomic Characterization of Rectal Cancer  Patient-derived Organoids (P6-P19) | National Omics Data  Encyclopedia NODE | [https://www.biosino.org/node/ project/detail/OEP000599](https://www.biosino.org/node/project/detail/OEP000599) |

**Whole-exome sequencing and known driver gene filtering**

DNA degradation and contamination were assessed on 1% agarose gels, and DNA concentration was measured using the Qubit DNA Assay Kit on a Qubit 2.0 Fluorometer (Invitrogen, USA). For each sample, 0.6 mg of genomic DNA was used for DNA sample preparation. Sequencing libraries were prepared using the Agilent SureSelect Human All Exon kit (Agilent Technologies, CA, USA), and index codes were added. Sample clustering was performed on a cBot Cluster Generation System with the Hiseq PE Cluster Kit (Illumina), followed by sequencing on the Illumina Hiseq platform to generate 150 bp paired-end reads. Sequence reads were aligned to the human reference genome GRCh37 using BWA-MEM v0.7.8-r455(1), and bam files were processed to mark duplicates, realign indels, and recalibrate bases using Genome Analysis Toolkit (GATK) v3.8.0(2). Somatic mutations were identified using MuTect2 (involved in GATK v3.8.0).

Known driver gene mutations were filtered based on the following criteria: Variants with depth <10X were excluded; low-frequency variants in the 1000 Genomes Project were retained, while SNPs in dbSNP were excluded unless present in the COSMIC database; variants in intergenic, non-coding, intronic regions, and synonymous mutations were excluded; variants in genomic repeat regions were excluded; variants were filtered using scores from ljb23_sift, ljb23_pp2hvar, ljb23_pp2hdiv, and ljb23_mt: at least one database rating the variant as harmful or two or more as moderately harmful.

Filtered somatic mutations were compared against known driver genes from several sources: Cancer Gene Census (https://cancer.sanger.ac.uk/census/), Bert Vogelstein's 125 mut-driver genes(3), significantly mutated genes (SMG127) from pan-cancer data(4), and a comprehensive set of 435 driver genes identified through various detection methods(5).

**Materials Availability**

Distribution of organoids to third parties mandates the completion of a material transfer agreement and must be approved by the Ethical Committee and Institutional Review Board of Fudan University Shanghai Cancer Center. The use of organoids is contingent upon patient consent; if consent is withdrawn, any distributed organoid lines and derived materials must be promptly destroyed.

**CRC Patient Derived Organoid Culture Medium**

| Regent name | Company | Cat No. | Stock  solution | Solvent | Final  concentration |
| --- | --- | --- | --- | --- | --- |
| Advanced  DMEM/F12 | GIBCO | 12634-010 | — | 1 × | 1 × |
| HEPES | Gibco | 15630080 | 100 × | — | 1 × |
| GlutMAX | Gibco | 35050-061 | 100 × | — | 1 × |
| Penicillin/ streptomycin | Solarbio | P1400 | 100 × | — | 1 × |
| N2 | Gibco | 17502-048 | 50 × | — | 1 × |
| B27 | Gibco | 17504-044 | 100 × | — | 1 × |
| EGF | Sino Biological | 50482-MNCH | 500μg/mL | 0.1%BSA/ PBS | 50ng/mL |
| N-Acetyl-L- cysteine | Sigma-aldrich | A9165 | 500mM | ddH2O | 1mM |
| Nicotinamide | Sigma-aldrich | N0636 | 1M | ddH2O | 10mM |
| Normocin | invivogen | ant-nr-2 | 500 × | — | 1 × |
| Gentamicin/ AmphoteritinB | Gibco | R01510 | 500 × | — | 1 × |
| A83-01 | Tocris | 2939 | 5mM | DMSO | 500nM |
| Prostaglandin E2 | Sigma-aldrich | P6532 | 100μM | DMSO | 10nM |
| Gastrin | Sigma-aldrich | G9145 | 100μM | 0.1%BSA/ PBS | 10nM |
| SB202190 | Sigma-aldrich | S7067 | 30mM | DMSO | 3μM |
| R-spondin-1 | Sino Biological | 11083-HNAS | 50μg/mL | 0.1%BSA/ PBS | 500ng/mL |
| Noggin | Sino Biological | 50688-M02H | 10μg/mL | 0.1%BSA/ PBS | 100ng/mL |

**H&E, Immunohistochemistry, and Immunofluorescence Staining**

The primary antibodies used for immunohistochemistry and immunofluorescence were listed below:

| Target | Company | Cat No. | Dilution |
| --- | --- | --- | --- |
| Ki-67 | Cell Signaling  Technology | 9449 | 1:500 |
| CDX2 | Cell Signaling  Technology | 12306 | 1:500 |
| β-Catenin | Cell Signaling  Technology | 8480 | 1:100 |
| CK20 | Cell Signaling  Technology | 13063 | 1:500 |
| γH2AX | Abclonal | AP0687 | 1:100 |
| CD44 | Proteintech | 15675-1-AP | 1:200 |
| Bcl-2 | Cell Signaling  Technology | 15071 | 1:1000 |
| Cleaved  Caspase-3 | Cell Signaling  Technology | 9664 | 1:1000 |

**References**

1. Li H, Durbin R. Fast and accurate short read alignment with Burrows-Wheeler transform. Bioinformatics. 2009 Jul 15;25(14):1754–60.

2. McKenna A, Hanna M, Banks E, Sivachenko A, Cibulskis K, Kernytsky A, et al. The Genome Analysis Toolkit: a MapReduce framework for analyzing next-generation DNA sequencing data. Genome Res. 2010 Sep;20(9):1297–303.

3. Vogelstein B, Papadopoulos N, Velculescu VE, Zhou S, Diaz LA, Kinzler KW. Cancer genome landscapes. Science. 2013 Mar 29;339(6127):1546–58.

4. Kandoth C, McLellan MD, Vandin F, Ye K, Niu B, Lu C, et al. Mutational landscape and significance across 12 major cancer types. Nature. 2013 Oct 17;502(7471):333–9.

5. Tamborero D, Gonzalez-Perez A, Perez-Llamas C, Deu-Pons J, Kandoth C, Reimand J, et al. Comprehensive identification of mutational cancer driver genes across 12 tumor types. Sci Rep. 2013 Oct 2;3:2650.

**Supplementary Figures**

**Figure S1. Sanger sequencing results of CRC PDOs, related to Figure 2.**


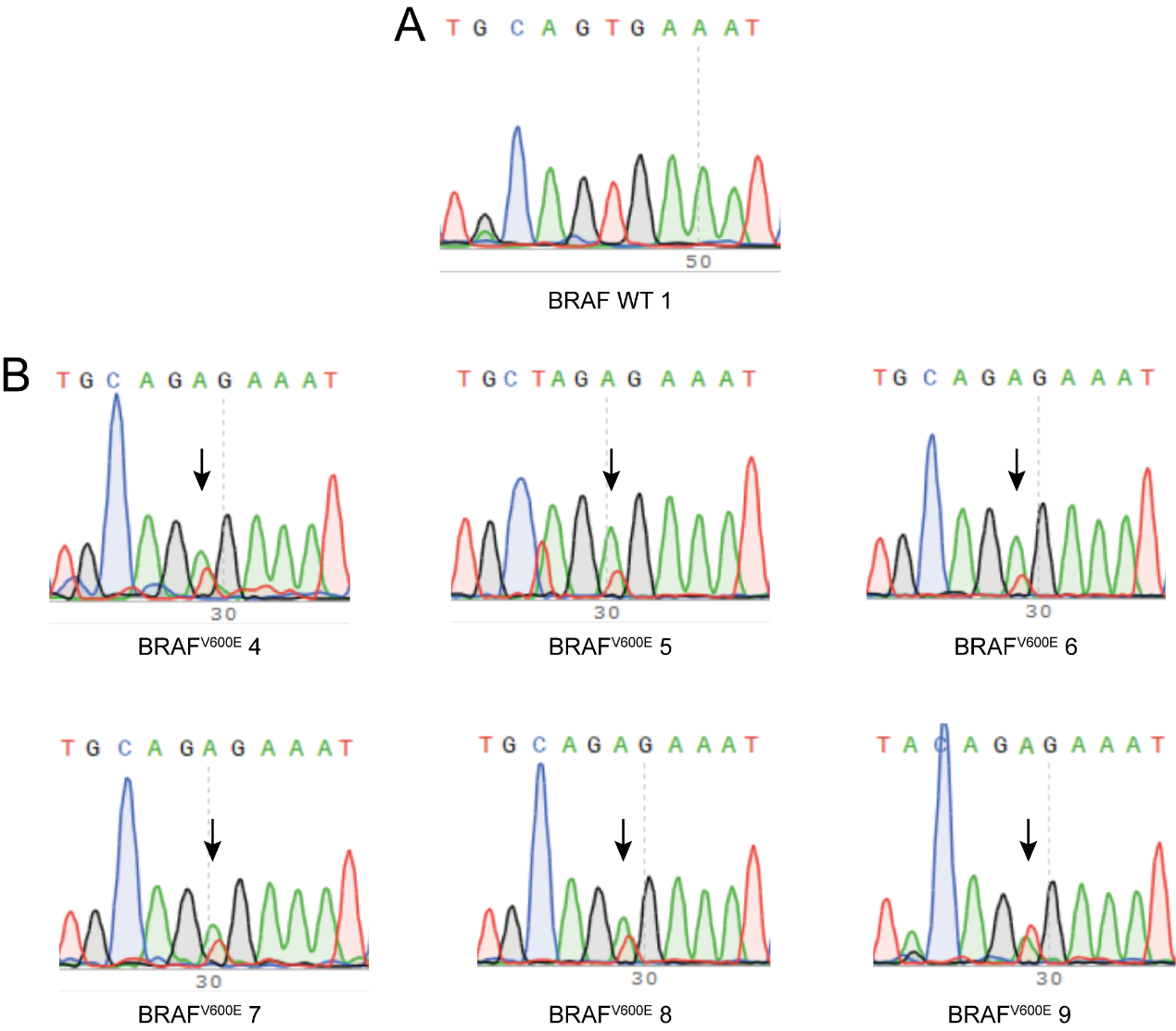


**Figure S1.** **Sanger sequencing results of CRC PDOs, related to Figure 2. A)** Sanger sequencing results of BRAF wild-type organoid. **B)** Sanger sequencing results of BRAF-mutant organoids (BRAF^V600E^ 4-9). The arrow indicated the BRAF^V600E^ mutation site.

**Figure S2. Representative brightfield images of organoids irradiated with escalating doses and immunofluorescent images of γH2AX foci 10 hours after 8 Gy radiation, related to Figure 3.**


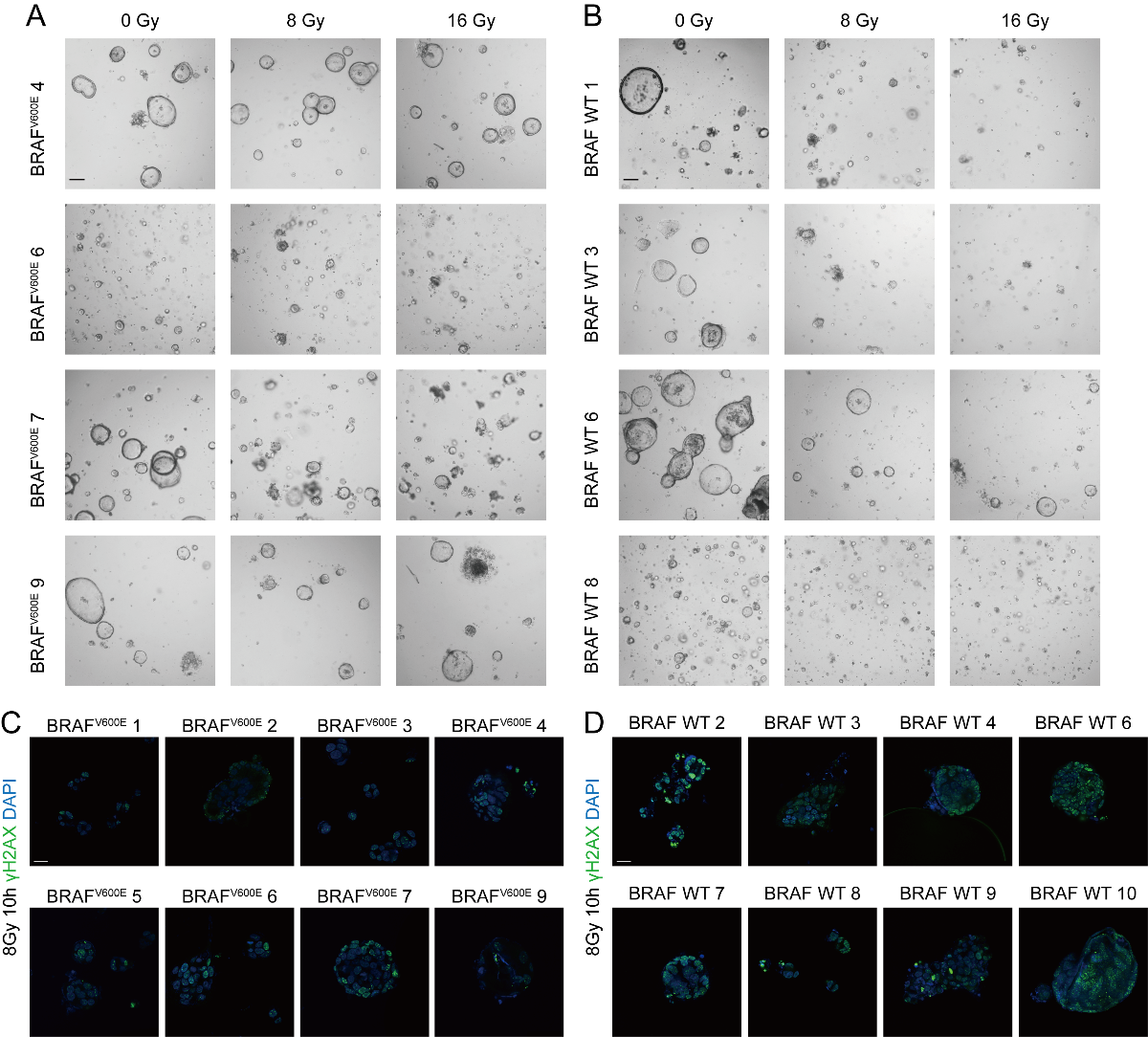


**Figure S2.** **Representative brightfield images of organoids irradiated with escalating doses and immunofluorescent images of γH2AX foci 10 hours after 8 Gy radiation, related to Figure 3. A)** Representative brightfield images of BRAF^V600E^-mutant organoids irradiated with escalating doses (0 Gy, 8 Gy, 16 Gy). Images were captured 6 days post radiation. Scale bar, 50 μm. **B)** Representative brightfield images of BRAF wild-type organoids irradiated with escalating doses (0 Gy, 8 Gy, 16 Gy). Images were captured 6 days post radiation. Scale bar, 50 μm. **C)** Representative high-magnified immunofluorescent images of γH2AX foci (green) in BRAF^V600E^-mutant organoids 10 hours post 8 Gy radiation. Scale bar, 20 μm. Nuclei were stained by DAPI. **D)** Representative high-magnified immunofluorescent images of γH2AX foci (green) in BRAF wild-type organoids 10 hours post 8 Gy radiation. Scale bar, 20 μm. Nuclei were stained by DAPI.

**Figure S3. Comparison of the size ratio 24 days after 8 Gy radiation between BRAF^V600E^-mutant and wild-type organoids.**


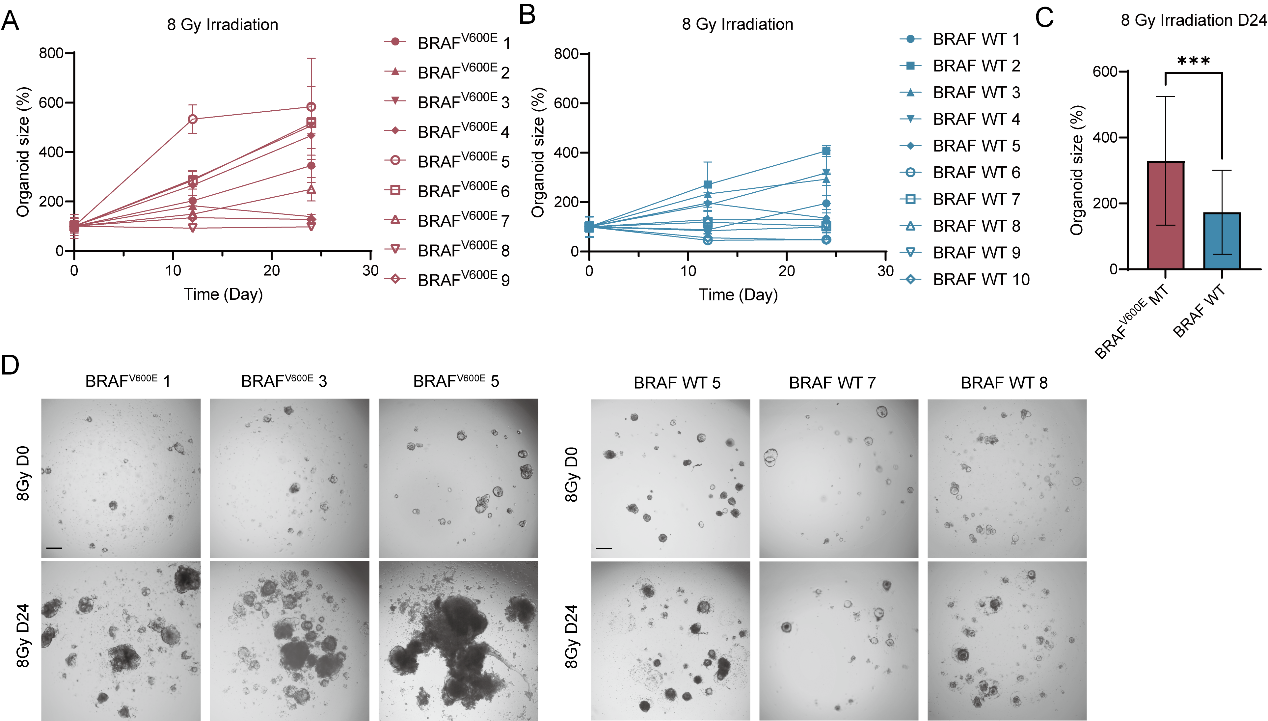


**Figure S3.** **Comparison of the size ratio 24 days after 8 Gy radiation between BRAF^V600E^-mutant and wild-type organoids. A)** BRAF^V600E^ mutant organoid size ratio 24 days following exposure to 8 Gy radiation. Data (mean ± SD) were collated from three different experiments. **B)** BRAF wild-type organoid size ratio 24 days following exposure to 8 Gy radiation. Data (mean ± SD) were collated from three different experiments. **C)** Size ratio of PDOS were compared between BRAF^V600E^-mutant and BRAF wild-type group. Size ratio was measured 24 days after 8 Gy radiation and normalized to organoid size on day 0. Statistical significance was analyzed by student’s t-test (329.9% vs173.4%， p=0.0004). Quantitation of organoid size ratio is presented as mean ± SD (n=9 in BRAF^V600E^ MT group, n=10 in BRAF WT group, each organoid with three different experiments)**. D)** Representative bright-field images of selected PDOs on day 0 and 24 post 8 Gy radiation (Left: BRAF^V600E^-mutant PDOs. Right: BRAF wild-type PDOs. Scale bar, 100 μm.).

**Figure S4. Comparison of regrowth between BRAF^V600E^-mutant and wild-type organoids after high-dose radiation.**


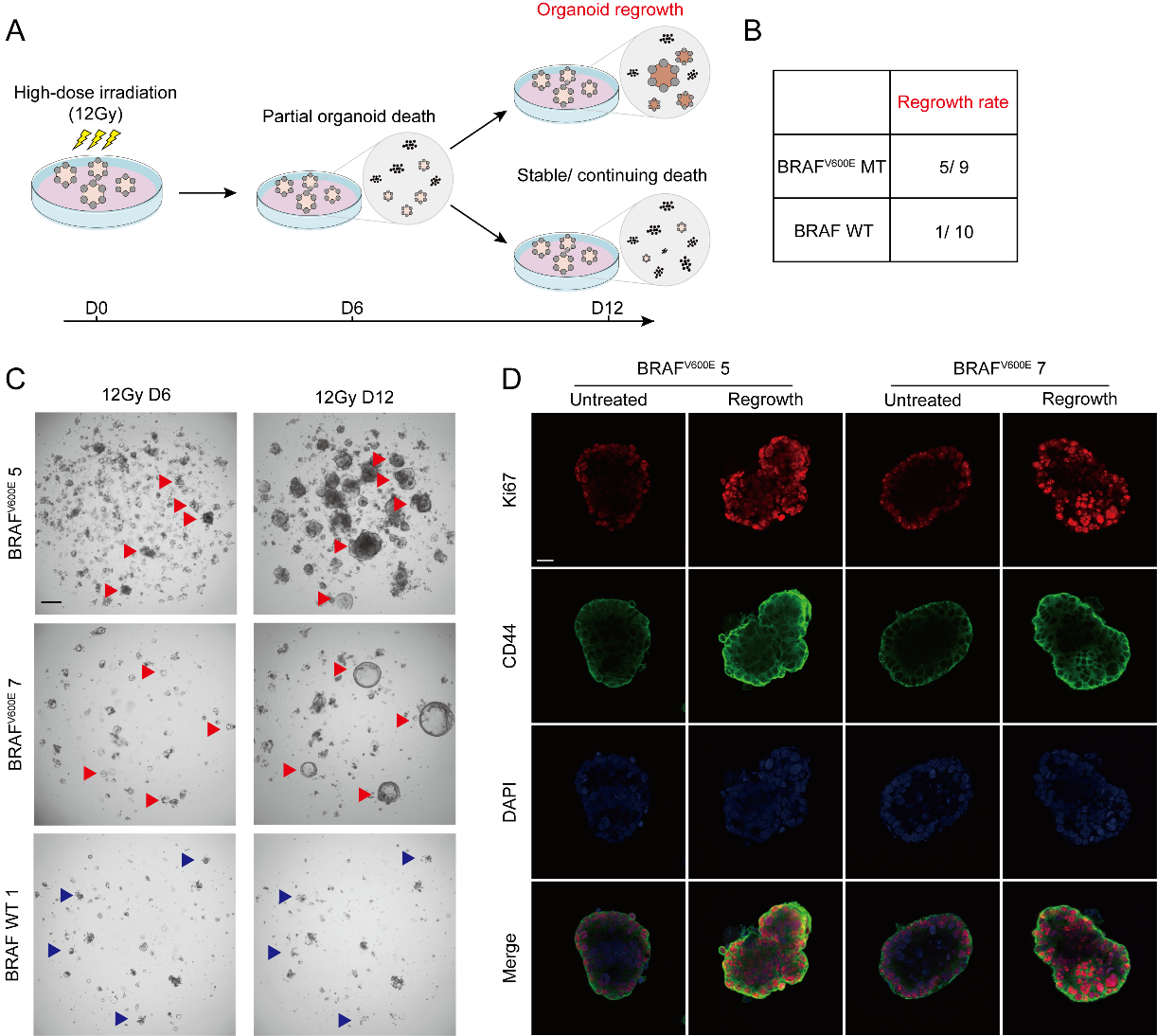


**Figure S4.** **Comparison of regrowth between BRAF^V600E^-mutant and wild-type organoids after high-dose radiation. A)** Diagram representing the regrowth phenomenon in organoids after receiving a high dose (12 Gy) of radiation. **B)** The proportion of BRAF^V600E^-mutant and BRAF wild-type organoids exhibiting the regrowth phenomenon. **C)** Representative bright-field images of organoids exhibiting the regrowth phenomenon (Left: images showing organoids with partial cell death on day 6 after receiving 12 Gy radiation. Right: images showing organoids with partial regrowth on day 12 after receiving 12 Gy radiation). Red arrows indicated organoids with regrowth, and blue arrows indicated organoids without regrowth. Scale bar, 100 μm. **D)** Representative immunohistochemistry staining of Ki67 (red) and CD44 (green) in two untreated organoids and their respective regrown organoids after receiving 12 Gy radiation. Scale bar, 20 μm. Nuclei were stained with DAPI.

**Figure S5. Radiosensitizing effects of BRAF inhibitors in BRAFV600E mutant CRC.**


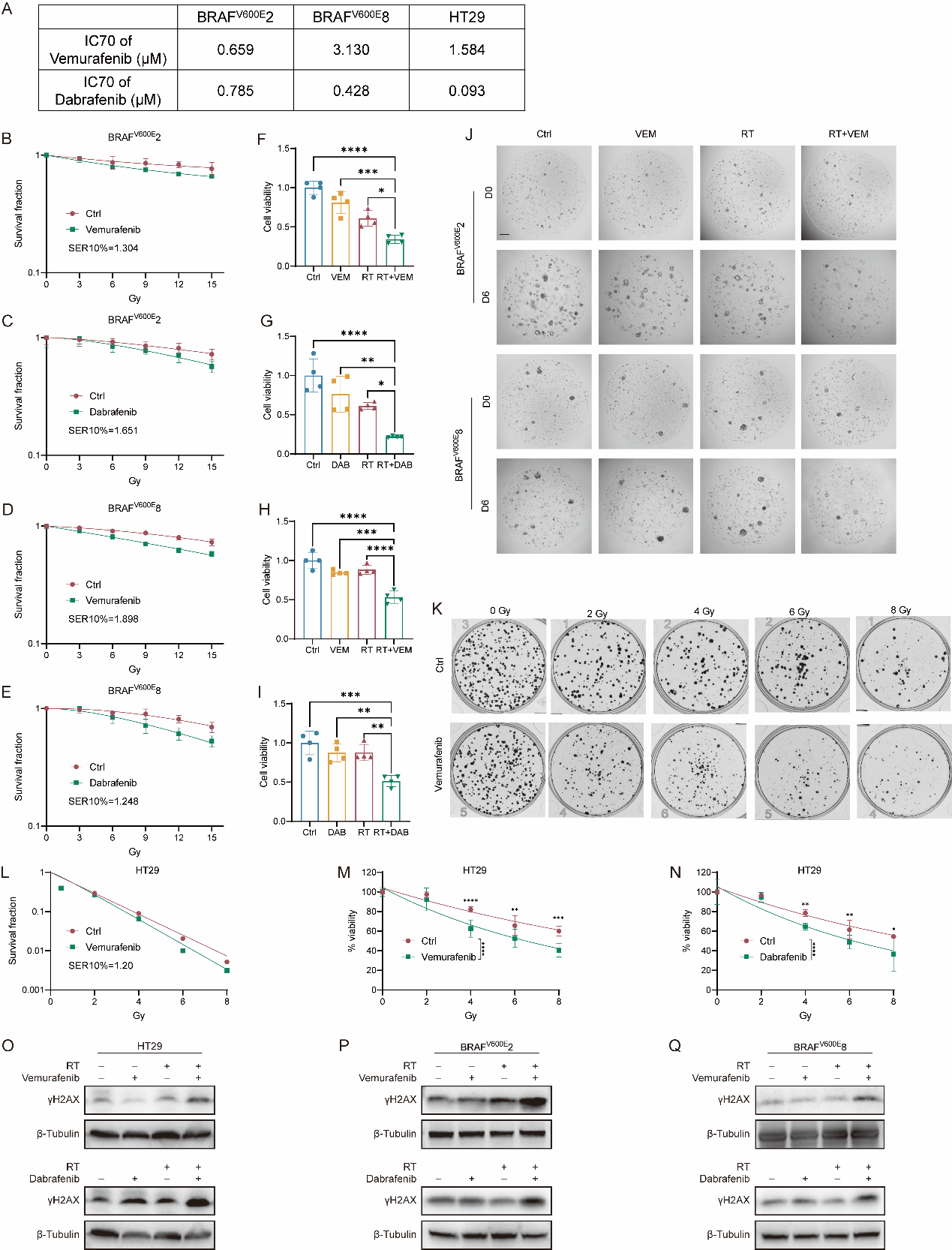
**Figure S5. Radiosensitizing effects of BRAF inhibitors in BRAFV600E mutant CRC. A)** IC70 concentrations of BRAF inhibitors in HT29 and BRAF^V600E^ mutant organoids. **B-E)** Dose survival curves BRAF^V600E^ mutant organoids treated with radiotherapy with or without BRAF inhibitors. Data (mean ± SD) were collated from three different experiments. Sensitizer enhancement ratios (SER10%) were demonstrated. **F-J)** Cell viability of organoids treated with radiotherapy, BRAF inhibitor and combined therapy. Cell viability was measured 6 days after treatments. Statistical significance was analyzed by one-way ANOVA. Data represent mean± SD (n=4 per group). **K, L)** Colony formation assay and dose survival curve of HT29 cells treated with increasing dose of radiotherapy, with or without Vemurafenib Treatment. Data (mean ± SD) were collated from three different experiments. Sensitizer enhancement ratios (SER10%) were demonstrated. **M, N)** Relative viability of HT29 cells treated with increasing dose of radiotherapy, with or without BRAF inhibitors. Statistical significance was analyzed by two-way ANOVA. Data represent mean± SD (n=4 per group). **O-Q)** γH2AX expression in HT29 and BRAF^V600E^ mutant organoids treated with radiotherapy, BRAF inhibitor and combined therapy.

**Figure S6. Impact of BRAF^V600E^ mutation on 5FU resistance and synergistic effect with BRAF Inhibitors.**


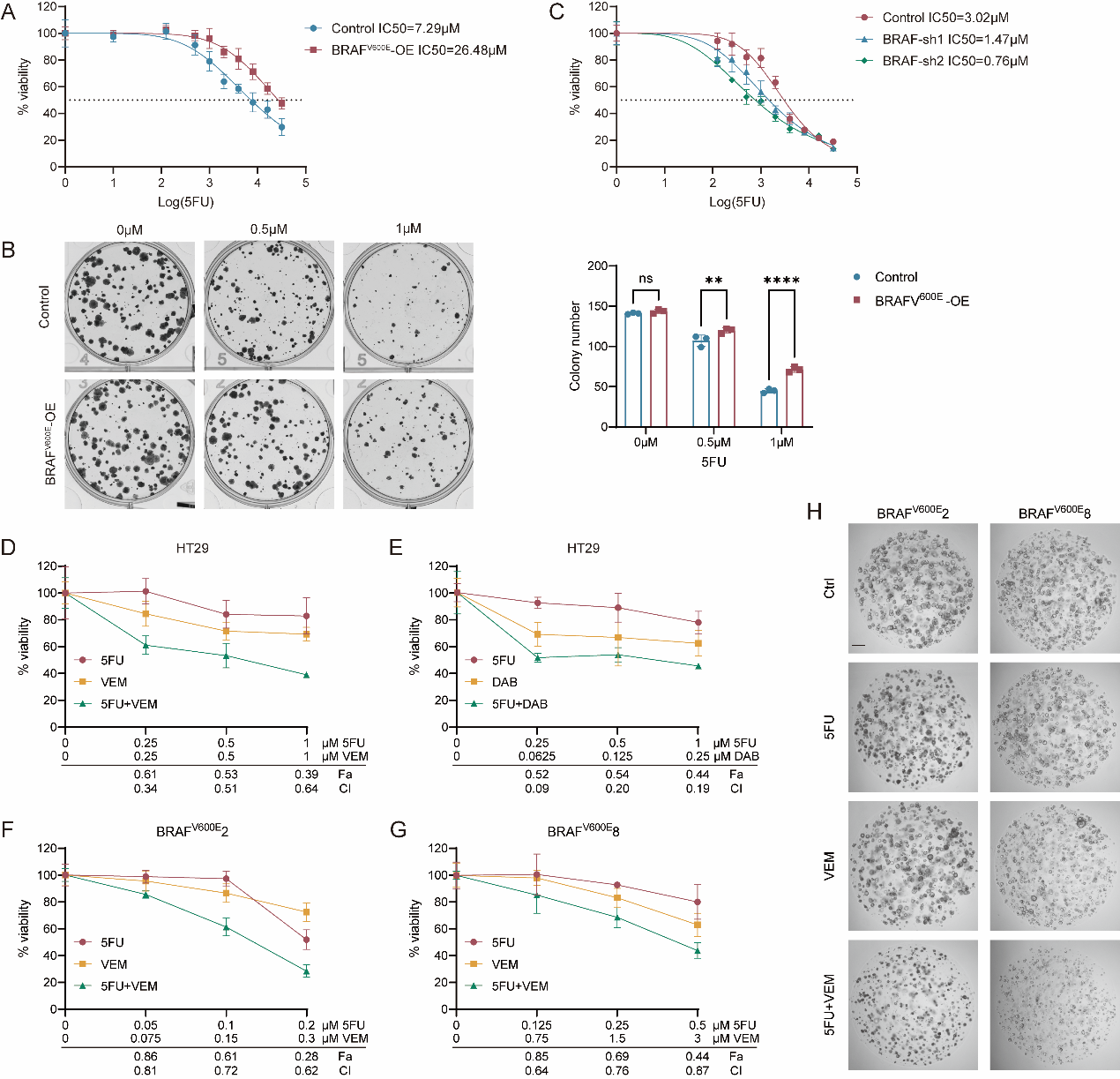


**Figure S6. Impact of BRAF^V600E^ mutation on 5FU resistance and synergistic effect with BRAF Inhibitors. A)** 5FU dose-response curves for BRAF^V600E^-OE and control Caco-2 cells. Data represent mean± SD (n=4 per group). **B)** Colony formation assay comparing the growth of BRAF^V600E^-OE and control Caco-2 cells under different 5FU concentrations. Data (mean ± SD) were collated from three different experiments. Statistical significance was analyzed by two-way ANOVA. **C)** 5FU dose-response curves for BRAF-knockdown (BRAF-sh1, BRAF-sh2) and control HT29. Data represent mean± SD (n=4 per group). **D, E)** Survival fractions of HT29 cells after exposure to gradient concentrations of 5FU in combination with Vemurafenib or Dabrafenib. Data represent mean± SD (n=4 per group). **F, G)** Survival fractions of BRAF^V600E^ mutant organoids after exposure to gradient concentrations of 5FU with Vemurafenib. Data represent mean± SD (n=4 per group). **H)** Representative brightfield images of BRAF^V600E^ mutant organoids treated with 5FU, Vemurafebin and combined therapy. Scale bar, 100 μm.

**Figure S7. Comparison of histological consistency between PDOX tissue and the original tissue and the toxicity responses in different treatment groups, related to Figure 6.**


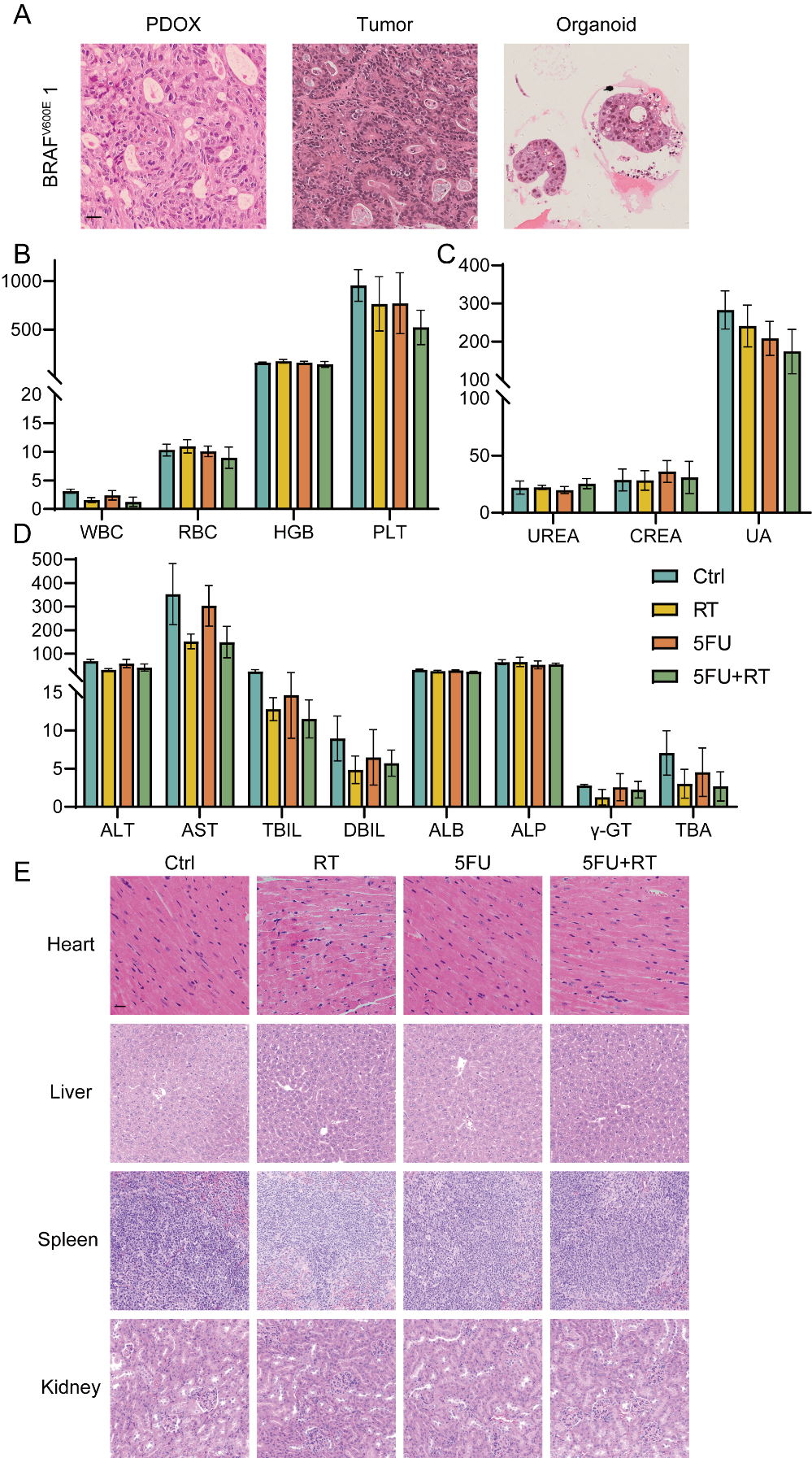


**Figure S7.** **Comparison of histological consistency between PDOX tissue and the original tissue and the toxicity responses in different treatment groups, related to Figure 6. A)** H&E staining of PDOX tissue isolated from untreated mouse comparing with BRAF^V600E^ 1 patient’s tumor tissue and corresponding organoid. Scale bar, 20 μm. **B)** The number of WBC, RBC, HGB and PLT in peripheral blood were compared in different treatment groups. Quantitation is presented as mean ± SD (n=5 per group)**. C)** The levels of kidney function-related indicators were compared among different treatment groups. Quantitation is presented as mean ± SD (n=5 per group). **D)** The levels of liver function-related indicators were compared among different treatment groups. Quantitation is presented as mean ± SD (n=5 per group). **E)** H&E staining of mean organs of PDOX mice in different treatment groups. Scale bar, 20 μm.
