# Supplementary figures and images for: Unveiling radiobiological traits and therapeutic responses of BRAFV600E-mutant colorectal cancer via patient-derived organoids
Source: J Exp Clin Cancer Res. 2025 Mar 11;44:92. doi: 10.1186/s13046-025-03349-z (PMC11895145; doi:10.1186/s13046-025-03349-z)

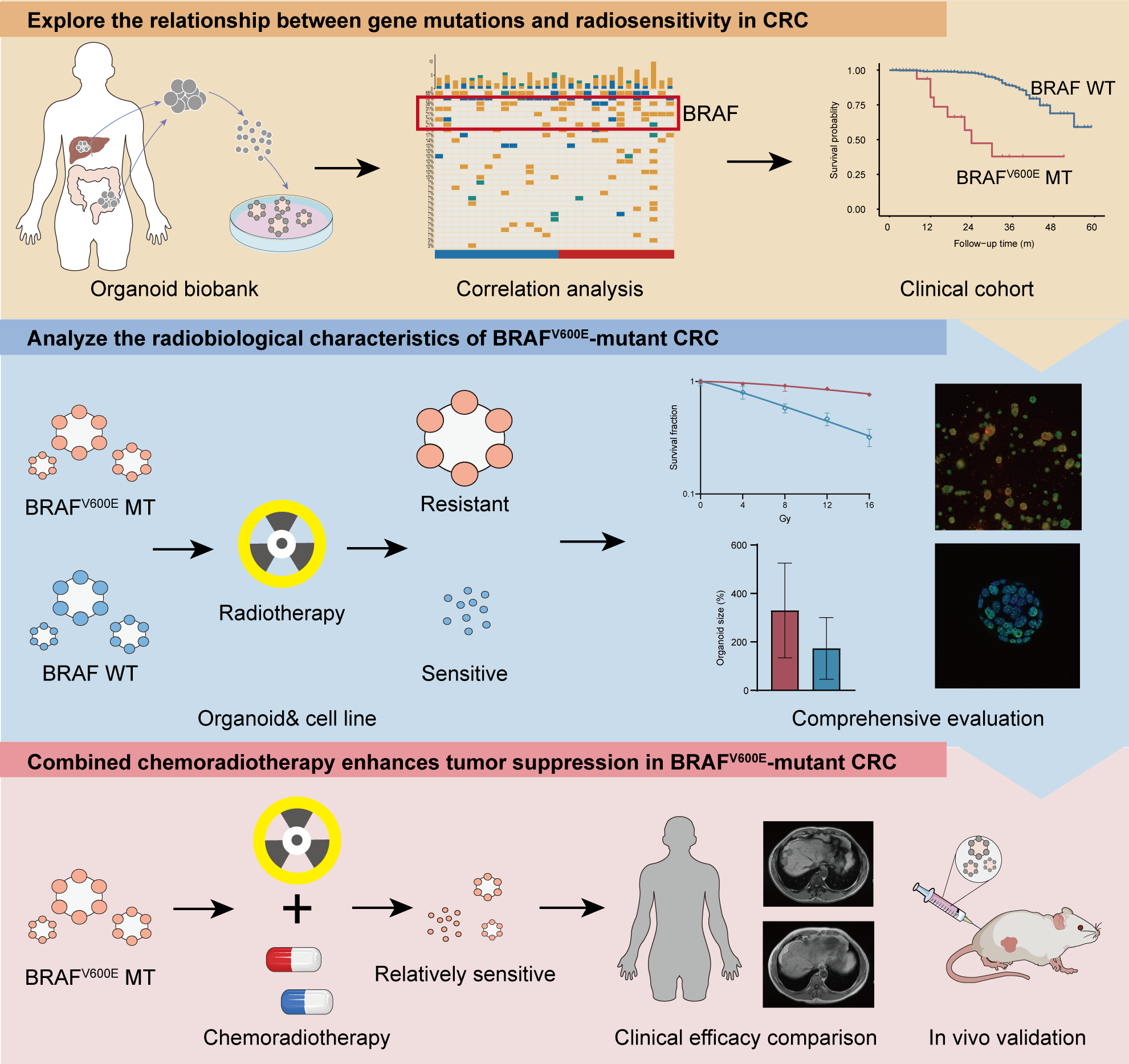

Supplement: Supplementary file 3 — Supplementary Material 3 [file 13046_2025_3349_MOESM3_ESM.tif]
